# Establishment of a Non-transgenic Iron-Biofortified Rice Line Using a Novel *HRZ1* Mutation

**DOI:** 10.1186/s12284-026-00897-6

**Published:** 2026-03-13

**Authors:** Akihiro Saito, Junya Kumano, Masataka Suzuki, Kento Nakamura, Hiromi Ichinokawa, Arata Higashimoto, Mai Kato, Kei Shimada, Sachiho Koshika, Satomi Nakayama, Nanami Kawano, Shunta Nishino, Takehiro Kobayashi, Haruka Nakamura, Kurumi Yamanaka, Ayane Konno, Rina Shimokawa, Ryoma Sugano, Shuhei Mukaida, Hayate Hata, Takuji Ohyama, Yusuke Shikanai, Toshihiro Kumamaru, Shimpei Uraguchi, Toru Fujiwara, Kyoko Higuchi

**Affiliations:** 1 Laboratory of Biochemistry in Plant Productivity, Department of Agricultural Chemistry, Tokyo University of Agriculture, Sakuragaoka1-1-1., Setagaya-ku, Tokyo, 156-8502 Japan; 2 Faculty of Agriculture, Kyushu University, 744 Motooka Nishi-ku, Fukuoka, 819-0395 Japan; 3 Laboratory of Plant Nutrition, Graduate School of Horticulture, Chiba University, Matsudo-city, 271-8510 Chiba Japan; 4 Laboratory of Plant Nutrition and Fertilizers, Department of Applied Biological Chemistry, Graduate School of Agricultural and Life Sciences, The University of Tokyo, Bunkyo-ku, Tokyo, 113-8657 Japan

**Keywords:** Fe-biofortified plant, Fe uptake and accumulation, Fe-deficiency anemia, HRZ1, Rice (*Oryza sativa*), GRAS-Di

## Abstract

Iron (Fe) deficiency anemia is a significant public health problem worldwide. The development of Fe biofortification in staple food crops, such as rice, through non-transgenic methods is highly anticipated to enhance broad applicability. In this study, we isolated a high Fe-accumulating mutant (*tetsu*) from an *N*-methyl-*N*-nitrosourea (MNU) mutagenized ‘Taichung-65 (T65)’ rice population. The *tetsu* mutant accumulated more than 3-fold higher levels of Fe and significantly higher levels of manganese (Mn) and nickel (Ni) in the shoot than the wild-type (WT) T65, whereas the levels of toxic heavy metals such as cadmium (Cd), lead (Pb), and cobalt (Co) were comparable to those of the WT. In both polished and brown rice of the *tetsu* mutant, Fe increased by approximately 2-fold, and Zn and Cu also significantly increased compared with those in T65. Perls’ staining revealed that Fe localization in rice grains was not limited to the outer layers and scutellum, but also extended into the endosperm of the *tetsu* mutant. Concomitant with high Fe accumulation, the *tetsu* mutant showed remarkable tolerance to alkaline Fe-deficient soil conditions. Genotyping by Random Amplicon Sequencing-Direct (GRAS-Di) analysis revealed a novel nonsense mutation in the *Hemerythrin motif-containing Ring Zinc-finger protein 1* (*HRZ1*) gene in the *tetsu* genome, which is known to govern the negative regulation of the Fe deficiency response and is crucial for normal development. The homozygous *tetsu* mutation leads to a substantial increase in shoot Fe content, alongside the upregulation of several genes related to Fe uptake and translocation, without causing serious adverse effects on growth. To utilize this novel mutation in Fe-biofortified rice breeding, we created recombinant inbred lines (RILs) derived from crosses between the *tetsu* mutant and ‘Asamurasaki,’ a nutrient-rich black rice cultivar. During the breeding process, we successfully selected RILs that exhibited normal growth and fertility, resulting in the development of non-transgenic Fe-biofortified rice lines with various waxy/glutinous properties and polyphenol content in brown rice for versatile applications. These results indicate that the identified novel *HRZ1* mutation is a valuable target for engineering non-transgenic Fe-biofortified rice cultivars with various beneficial traits.

**Supplementary Information:**

The online version contains supplementary material available at 10.1186/s12284-026-00897-6.

## Background

Fe is an essential element for most living organisms, coordinating with many enzymes responsible for electron transfer and redox reactions to produce energy, mainly through photosynthesis and respiration (Kermeur et al. 2023). Fe deficiencies are among the most prevalent micronutrient deficiencies worldwide, affecting 2 billion people and causing more than 0.8 million deaths annually (Murgia et al. 2012; WHO 2019). Biofortification is a cost-effective and sustainable approach that aims to solve this problem by boosting the micronutrient content of crops (Connorton and Balk 2019). Rice (*Oryza sativa*) is a staple food for over 3.5 billion people and comprises approximately 23% of the calories consumed worldwide (Khush 2003; Hackl et al. 2019; Kermeur et al. 2023). The development of Fe biofortified rice is a solution to the global problem of Fe deficiency anemia (Hackl et al. 2019). In addition to Fe, copper (Cu) and zinc (Zn) are essential micronutrients for human health, playing critical roles in immune function, growth, and development. Because staple crops are major dietary sources of these micronutrients, biofortification strategies should consider the accumulation of Cu and Zn as well as Fe (White and Broadley 2009).

Rice plants acquire Fe from soil as ferrous Fe (Fe^2+^) through OsIRT1, OsIRT2, and OsNRAMP1 (Ishimaru et al. 2006), and as the phytosiderophore 2′-deoxymugineic acid (DMA)–Fe^3+^ complex (Takagi 1976; Curie et al. 2000) through Yellow Stripe 1 (YS1)/YSL transporters, such as OsYSL15 (Curie et al. 2001, 2009; Inoue et al. 2008, 2009; Suzuki et al. 2021). DMA synthesis in rice is catalyzed by nicotianamine synthase (NAS) to produce the Fe^2+^ chelator nicotianamine (NA) from three molecules of methionine (Higuchi et al. 1999, 2001), followed by the conversion of NA to DMA via nicotianamine aminotransferase (NAAT) and deoxymugineic acid synthase (DMAS) (Takahashi et al. 1999, 2001; Cheng et al. 2007; Inoue et al. 2008; Bashir et al. 2017). A transporter of mugineic acid (MA) family phytosiderophores (TOM1) is responsible for transporting DMA from the roots into the rhizosphere (Nozoye et al. 2011). Almost all the genes involved in above Fe uptake mechanism are strongly upregulated in response to Fe deficiency (Kobayashi et al. 2014).

After Fe absorption, OsYSL2 and other YSLs support the long-distance translocation of Fe as MA–Fe^3+^ and NA–Fe^2+^ complexes from the roots to shoots (Ishimaru et al. 2010; Koike et al. 2004; Aoyama et al. 2009; Senoura et al. 2017). Additionally, FERRIC REDUCTASE DEFECTIVE LIKE 1 (OsFRDL1) is a transporter involved in the efflux of citrate into the xylem for efficient Fe translocation (Ariga et al. 2014; Yokosho et al. 2016). Other transporters are also involved in Fe transport/translocation: OsPEZ1/2, which facilitates Fe transport by effluxing phenolic compounds with Fe-chelating activity (Bashir et al. 2011a; Ishimaru et al. 2011a, b), EFFLUX TRANSPORTER OF NA 1/2 (OsENA1/2); and OsTOMs NA efflux transporters (Nozoye et al. 2011, 2019). Fe transported into the cell is further distributed to organelles by the vacuolar Fe transporter OsVIT1/2 (Zhang et al. 2012; Che et al. 2021) and the mitochondrial Fe transporter MIT1 (Bashir et al. 2011b). The vacuolar MA transporter, OsVMT, also plays a key role in controlling the subcellular partitioning of MAs, thereby regulating metal translocation to grains (Che et al. 2019). Excess Fe within cells is sequestered in the vacuoles and the Fe-storage protein ferritin (OsFER1 and OsFER2) (Stein et al. 2009; Briat et al. 2010).

Attempts have been made to generate Fe-enhanced rice plants by controlling the expression of individual genes or a combination of genes responsible for the transport, translocation, and accumulation of Fe using genetic engineering techniques (Masuda et al. 2020; Kawakami and Bhullar 2021). In addition to these Fe transport- and storage-related genes, manipulation of the transcriptional and post-translational regulators of Fe accumulation could potentially be utilized for Fe biofortification. For example, overexpression of the transcription factors IDEF1 and IRON-REGULATED TRANSCRIPTION FACTOR 2 (OsIRO2) or IRON-REGULATED TRANSCRIPTION FACTOR 3 (OsIRO3) knockout enhances Fe-deficiency responses and increases Fe acquisition. Here, the transcription factors IDEF1 and IDEF2, located upstream of the Fe signaling pathway in rice, positively regulate the early Fe-deficiency response, primarily for most genes involved in DMA–Fe (III) and Fe^2+^ uptake (Kobayashi et al. 2007; Ogo et al. 2008). The basic helix-loop-helix (bHLH) transcription factor OsIRO2 positively regulates Fe deficiency–induced gene expression under the control of IDEF1. The bHLH gene OsIRO3, a homolog of POPYE (PYE) in Arabidopsis, negatively regulates Fe deficiency–inducible genes (Ogo et al. 2007, 2011; Zheng et al. 2010; Wang et al. 2020).

As post-translational regulators, the hemerythrin motif–containing RING zinc-finger proteins (HRZ1 and HRZ2), which function as E3-ubiquitin ligases and homologs of BRUTUS (BTS) in Arabidopsis, negatively control most known Fe deficiency–inducible genes, including the bHLH transcription factors OsIRO2 and OsIRO3, by degradation via the 26 S proteasome. Notably, HRZs contain histidine, histidine, and glutamic acid (HHE) domains with direct Fe- and Zn-binding properties and are thus considered promising candidates for Fe sensors in rice cells (Kobayashi et al. 2013, 2014, 2022; Pullin et al. 2025). HRZs also negatively regulate the expression of OsIMA1 and OsIMA2, which are rice orthologs of the small signaling peptide IRON MAN (IMA)/Fe UPTAKE INDUCING PEPTIDE (FEP) found in Arabidopsis (Grillet et al. 2018), at the transcriptional level via an unknown pathway (Kobayashi et al. 2021). *HRZ2* knockdown reportedly enhances Fe deficiency tolerance and Fe accumulation (Kobayashi et al. 2013, 2022). Regarding HRZ1, detailed phenotypic analysis of the previous *hrz1* mutant had not been advanced due to its poor growth phenotype. Recently, several transformants and genome-edited rice plants that accumulate the mutated HRZ1 protein lacking the C-terminal region have been created using a transgenic strategy and a genome-editing technique, and exhibited enhanced Fe-deficiency responses, similar to the suppression of *HRZ2* (Shinkawa et al. 2025). However, since all of these have undergone genetic modification, their yield potential and practicality for use as Fe-biofortified rice have not been examined.

In this study, we developed practical Fe-biofortified rice lines by isolating a rice mutant with high Fe content in the shoot from a screening of 3,000 MNU-mutagenized mutant lines. We identified a novel nonsense mutation in the fourth exon of the *HRZ1* gene, which corresponds to the beginning of the second hemerythrin domain of HRZ1 proteins. This mutation exhibited a pronounced phenotype characterized by Fe accumulation throughout the shoot, including in leaves and grains. This is the first report to demonstrate that the discovered *HRZ1* mutation is beneficial for producing non-transgenic, practical, biofortified rice plants.

## Results

### Isolation of a Rice Mutant *tetsu* with high Fe Content in Shoots and Xylem Sap

**Fig. 1:**
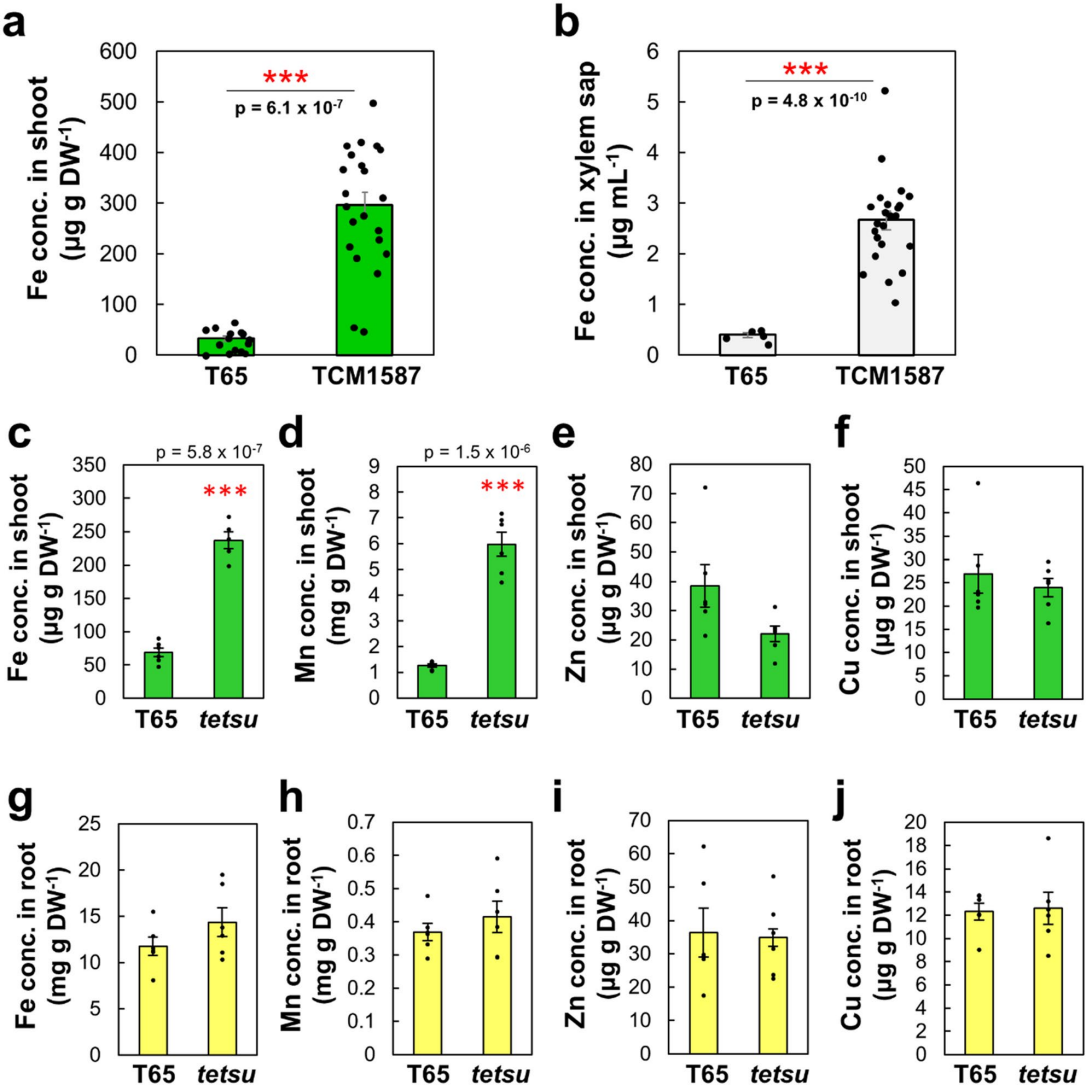
*tetsu* mutant accumulates high concentrations of Fe and Mn in the shoot. Fe concentrations in shoots (**a**) and xylem sap (**b**) of ‘Taichung-65’ (T65) and TCM1587 mutant. Plants were grown for 37 days on soil in an air-conditioned greenhouse (24 ± 3 °C) under natural light. Each column is the mean ± standard error with significant differences between T65 and TCM1587 determined using a Student’s *t*-test (*n* = 15 and *n* = 22 for shoots of WT and TCM1587, respectively, and *n* = 6 and *n* = 24 for xylem saps of T65 and TCM1587, respectively). Fe, Mn, Zn, and Cu concentrations in shoots (**c**, **d**, **e**, and **f**) and roots (**g**, **h**, **i**, and** j**) of T65 and *tetsu* mutant selected from TCM1587. Plants were grown for 26 days on soil in the growth chamber at 28 °C and a light intensity of 200 µmol photon m^− 2^ s^− 1^ under a 14 h light/10 h dark cycle. Each column is the mean ± standard error with significant differences (***P < 0.001) between T65 and *tetsu* using Student’s *t*-test (*n* = 4–5)

A rice mutant that accumulated high amounts of Fe was screened from an MNU-mutagenized T65 mutant population (TCM lines). As part of the primary screening, we performed an ionome analysis of the shoots and xylem sap of 21-day-old seedlings of the mutant population, as previously reported (Tanaka et al. 2018). Of the 2,704 germinated TCM lines, one mutant line, TCM1587, had higher Fe concentrations in both the shoot and xylem sap (9- and 6.5-fold on average, respectively) than T65 (Fig. 1a, b). As a secondary screening step, prompted by the large variation in Fe content within TCM1587, we selected one progeny line with a fixed high Fe accumulation trait, showing 3- and 4-fold increases in shoot Fe and Mn, respectively (Fig. 1c, d). Zn and Cu concentrations in shoots (Fig. 1e, f) and Fe, Mn, Zn, and Cu concentrations in roots (Fig. 1g–j) were not significantly different between T65 and the progeny line. Hereafter, we refer to this line as *Transporting Errors in TranSition metal Uptake (tetsu)*: *tetsu* means iron in Japanese.

### *tetsu* Mutant Does Not Absorb Excess Toxic Heavy Metals

Because the *tetsu* mutant accumulated higher concentrations of Fe and Mn than did T65, we assumed an increase in the accumulation of other heavy metals such as Ni, Co, Cd, and Pb. To clarify this, the germinated seeds of this mutant were grown in soil containing low concentrations of Ni, Co, Cd, and Pb (10 mg kg soil^− 1^ for Ni and 1 mg kg soil^− 1^ for Co, Cd, and Pb), and the amount of heavy metal absorption was measured (Fig. S1a, b). The results showed a 1.8-fold increase in the essential element Ni in the shoots and roots of the *tetsu* mutant compared with that in T65, which was mostly stored in the roots and minimally transferred to the shoots (Fig. S1d). Other non-essential heavy metals (Co, Cd, and Pb) accumulated equally in the *tetsu* mutant and T65, both in the roots and shoots (Fig. S1e–g), confirming that *tetsu* did not excessively accumulate these heavy metals. At the same time, even under heavy metal–contaminated soil conditions, Fe accumulation in the shoots of the *tetsu* mutant was approximately twofold higher than that in T65 (Fig. S1c).

### *tetsu* Mutant Accumulates Fe in Grains

**Fig. 2:**
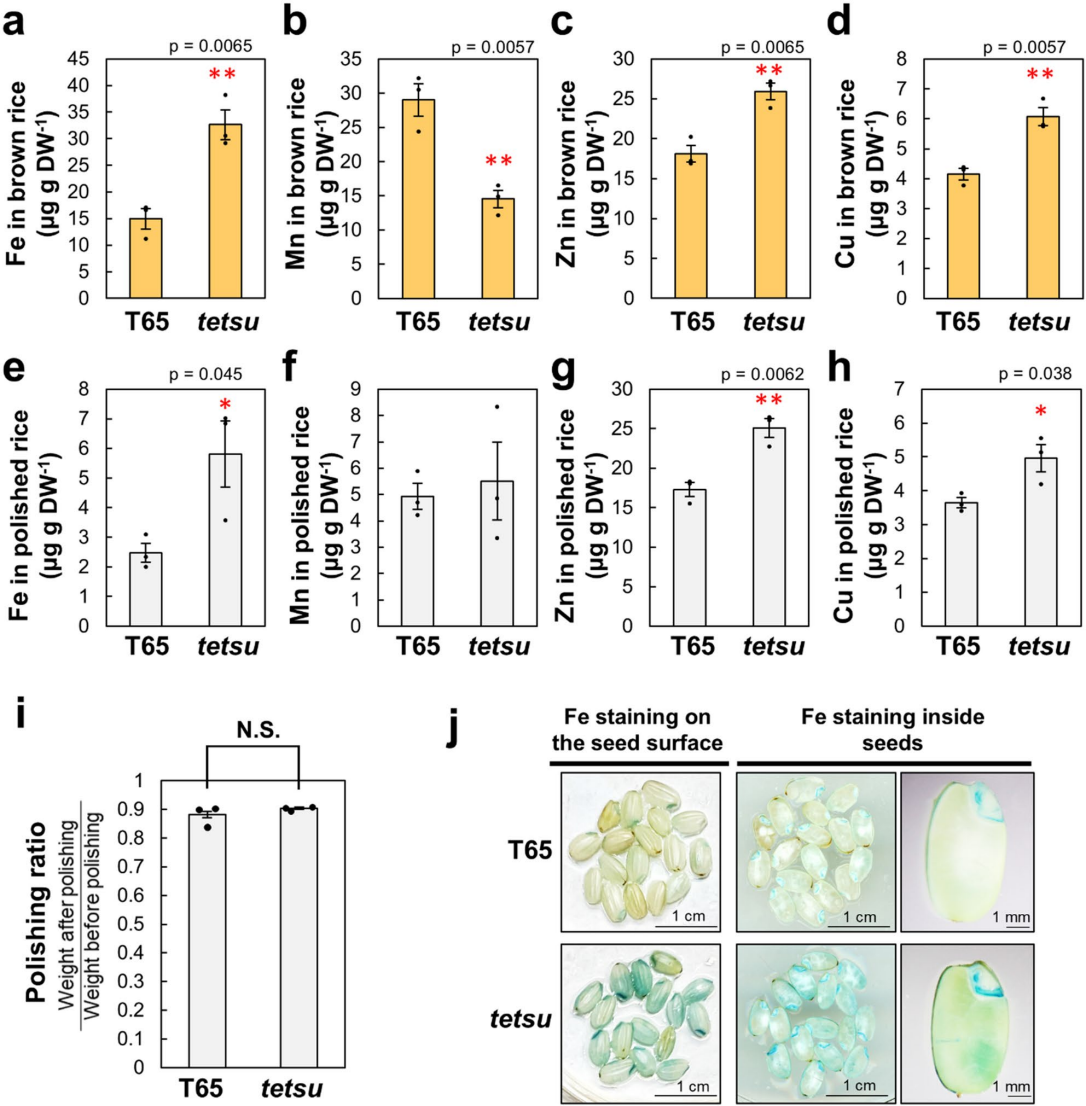
*tetsu* mutant seeds accumulate high levels of Fe, Zn, and Cu. Fe, Mn, Zn, and Cu concentrations in brown (**a**, **b**, **c**, and **d**) and polished (**e**, **f**, **g**, and **h**) T65 and *tetsu* mutant rice. T65 and *tetsu* mutant plants were grown on soil until fully ripe in a controlled greenhouse under natural light and supplemental light with a light intensity of more than 400 µmol m^− 2^ s^− 1^ during the day, with a 14 h light (28 °C)/10 h dark (25 °C) cycle. Each column is the mean ± standard error with significant differences (* *P* < 0.05, ** *P* < 0.01, *** *P* < 0.001) between T65 and *tetsu* using Student’s *t*-test (*n* = 3). The polishing rates of polished T65 and *tetsu* used for metal measurements did not significantly differ (**i**). Fe localization images obtained from Perls’ staining on the seed the surface side and inside (**j**)

Next, we measured the metal content of brown and polished rice. In the brown rice of the *tetsu* mutant, Fe increased more than 2-fold, Zn and Cu also increased significantly by over 40%, whereas Mn decreased significantly compared with that in T65 (Fig. 2a–d). In polished rice, the *tetsu* mutant exhibited approximately 2-fold higher Fe, 1.3-fold higher Zn, and 1.5-fold higher Cu than those in T65 (Fig. 2e–h). Because we prepared polished rice at the same milling ratio of 90% between T65 and *tetsu*, the amount of remaining bran did not affect this difference (Fig. 2i). Thus, we concluded that *tetsu* had significantly higher levels of Fe and moderately higher levels of Zn and Cu in the endosperm than did T65. To further verify the high Fe accumulation traits in grains, we analyzed Fe localization in rice grains using Perls’ staining. The stained images visually revealed that Fe was not limited to the outer layers (pericarp and aleurone layer) and scutellum, but also accumulated in the endosperm of the *tetsu* mutant compared with that in T65 (Fig. 2j).

### *tetsu* Mutant has Significant Alkaline Soil Tolerance

**Fig. 3:**
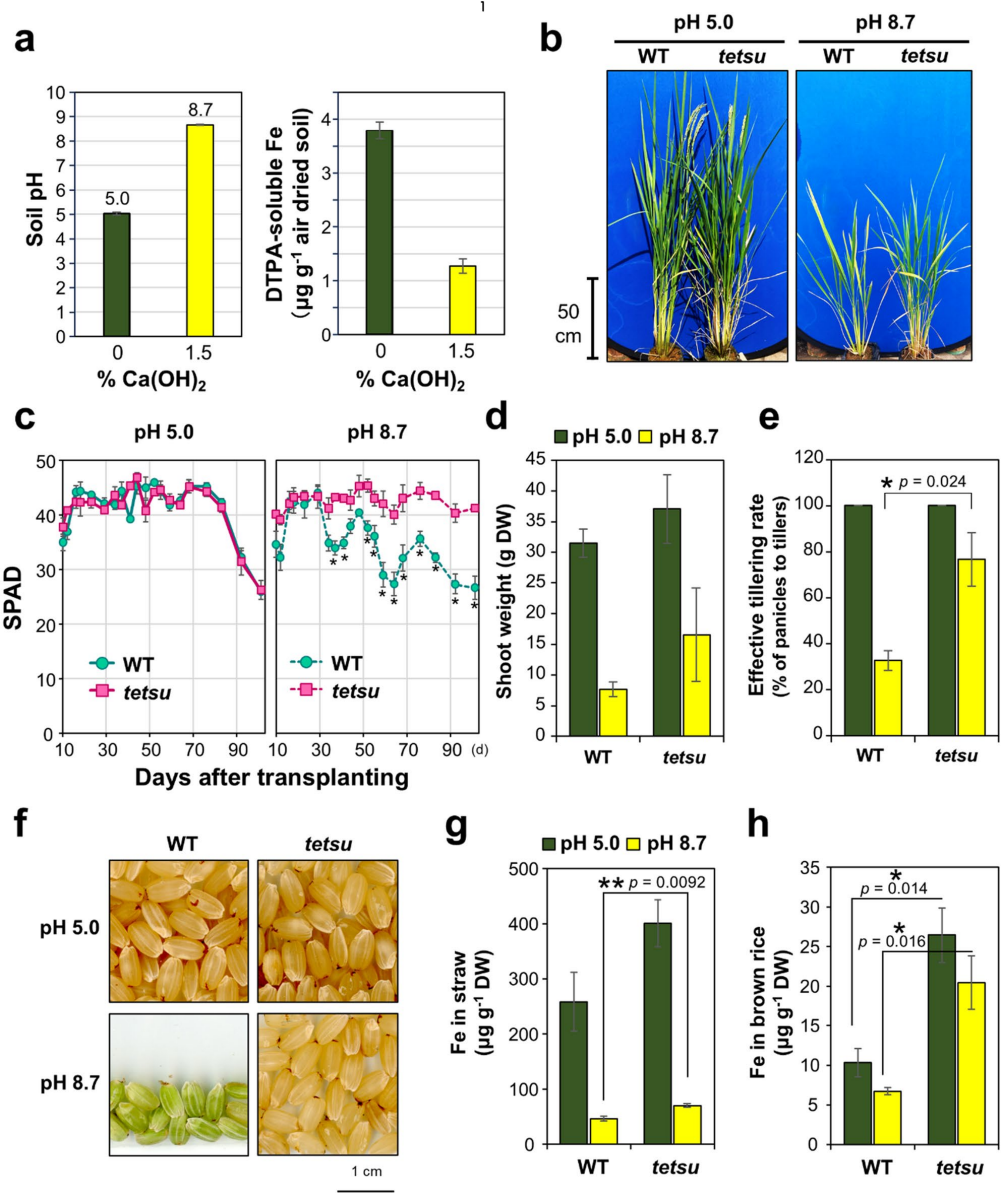
*tetsu* mutant tolerates Fe-deficient alkaline soil conditions, Soil pH and concentration of DTPA-extracted forms of Fe (**a**) in the soils used. Typical images of T65 and *tetsu* grown in soil at pH 5.0 and 8.7 during the heading period (**b**). Changes in SPAD values of plants grown in soils with different pH values (**c**). Young seedlings were pre-cultured in soil (pH 5.0) for 38 days and then transferred to soil at pH 5.0 or 8.7. The SPAD values were measured periodically for 101 days after transplantation. In soil with a pH of 5.0, leaves yellowed after 80 days owing to senescence during ripening. After harvest, the following data on yield and Fe content were collected: dry weight of shoots (**d**), effective tillering rate (**e**), image of harvested brown rice (**f**), and Fe concentrations in straw (**g**) and brown rice (**h**). Data represent the mean ± standard error with significant differences between T65 and *tetsu* using Student’s *t*-test (* *P* < 0.05, ** *P* < 0.01, *n* = 3)

High Fe accumulation in the shoots of the *tetsu* mutant was also expected to contribute to Fe deficiency tolerance. To confirm this possibility, Fe-deficiency tolerance was investigated in a calcareous alkaline soil environment using culture soil with calcium hydroxide [Ca(OH)_2_]. At a concentration of 1.5% (w/w) Ca(OH)_2_ in the rice culture soil (pH 5.0), soil pH increased to 8.7, and the amount of available Fe decreased to approximately 30% (Fig. 3a and Fig. S2), which is typical for calcareous Fe-deficient soils. The high pH persisted throughout cultivation; therefore, Ca(OH)_2_ was only mixed before planting.

A high Fe-containing F_3_ population backcrossed with the *tetsu* mutant to T65 was used to exclude the effects of mutations not involved in Fe accumulation in the *tetsu* mutant. After 38 days of pre-cultivation in pH 5.0 control soil, plants were transplanted into pH 5.0 or 8.7 soil for an additional 101 days. At pH 5.0, the appearance (Fig. 3b), SPAD value of the latest expanded leaves (Fig. 3c), shoot weight (Fig. 3d), and effective tillering rate (percentage of tillers that led to panicle formation) (Fig. 3e) were similar between WT and *tetsu* homozygous plants with no significant difference. Conversely, at pH 8.7, the WT showed significantly lower SPAD values in young leaves than did *tetsu* at 37 days after transplanting, and although transient recovery was observed, the SPAD values of WT continued to decrease until the end of cultivation (Fig. 3c). Meanwhile, *tetsu* maintained young leaf color at the same level as that at pH 5.0 during the cultivation period (Fig. 3c). Growth at pH 8.7 was equally reduced in both WT and *tetsu* compared with that at pH 5.0 under alkaline conditions (Fig. 3a, d), but the effective tillering rate was significantly higher than that in WT at pH 8.7 (Fig. 3e). At the end of cultivation (101 days after transplanting), seeds in WT were still green and immature, whereas many of the seeds in the *tetsu* mutant had almost reached full maturity (Fig. 3f). The Fe concentration in the shoots and brown rice was significantly higher in the *tetsu* mutant than that in WT plants (Fig. 3g, h).

Since both the WT and *tetsu* mutant plants had few seeds at pH 8.7, accurate analysis of yield components was not possible; therefore, we conducted a second trial to confirm the above results under milder alkaline conditions using 1.0% Ca(OH)_2_, where the soil pH was approximately 8.0, with the DTPA-Fe level significantly unchanged at the beginning of cultivation compared with that at pH 5.0 (Fig. S2). As shown in Fig. S3a, the grain weight of the *tetsu* mutant did not change between pH 5.0 and 8.0, whereas that of the WT significantly decreased to approximately 60% at pH 8.0. Total straw weight and effective tillering rate remained unchanged (pH 8.0) in the *tetsu* mutant, but were decreased in WT at pH 8.0 (Fig. S3b, c). The number of panicles showed no significant difference; however, that in WT tended to decrease at high pH, whereas the *tetsu* mutant showed no change (Fig. S3d). WT and the *tetsu* mutant did not significantly differ in the number of tillers, grain weight per panicle, and grain-filling rate (Fig. S3d–h).

Taken together, we confirmed that the *tetsu* mutant was tolerant to alkaline Fe-deficient conditions, maintaining source size (total straw weight) and effective tillering rate, resulting in higher yield in the *tetsu* mutant than in the WT under these conditions.

### High Fe Accumulation Trait in *tetsu* is Associated with a Novel Nonsense Mutation in *HRZ1*

**Fig. 4:**
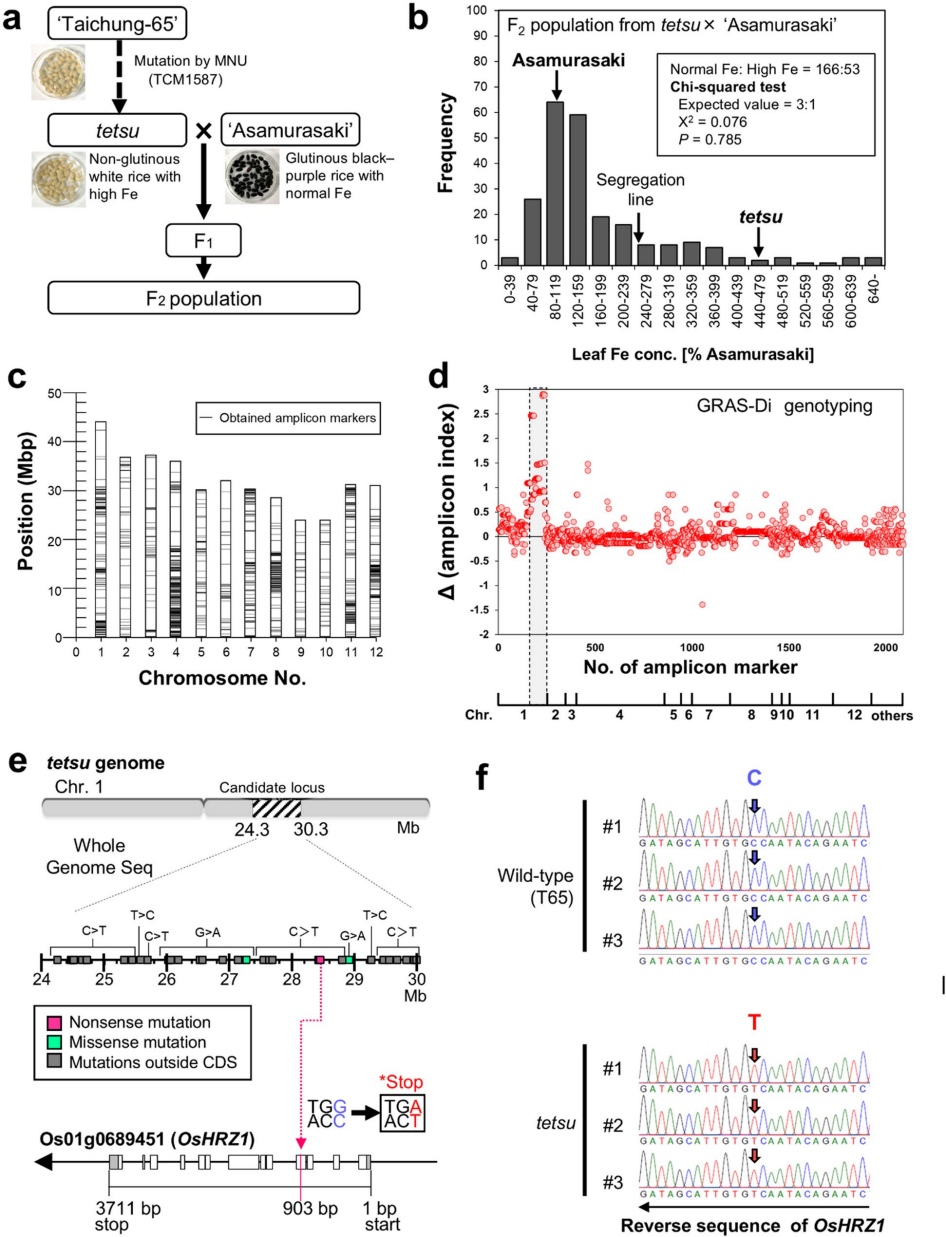
OsHRZ1 gene emerged as the causative gene for increased Fe content in the tetsu mutant. **a** Schematic diagram of the breeding steps to obtain F_2_ lines used for the genetic analysis. **b** Frequency distribution of Fe content in shoots of *tetsu* mutant, ‘Asamurasaki,’ and their F_2_ populations (*n* = 235). The chi-square test predicted that the causative gene was a single recessive gene. **c** The genomic location of the amplicon markers for distinguishing between genomes of *tetsu* mutants and ‘Asamurasaki.’ **d** Δ(amplicon index) was developed to visualize the genome position of the causal gene after GRAS-Di analysis for the F_2_ population. (**e**) Schematic diagram of a nonsense mutation found within the *OsHRZ1* gene in the candidate region (24.3–30.3 Mb) on chromosome 1 of the *tetsu* mutant. Details are shown in Table S1. **f** Mutations within the *OsHRZ1* gene present in the *tetsu* mutant reconfirmed using the Sanger sequencing of three independent plants. Arrows indicate the position of the mutated base (C to T)

Next, to identify the gene responsible for the high Fe accumulation of *tetsu*, we crossed the *tetsu* mutant with the Japanese glutinous black–purple rice cultivar ‘Asamurasaki’ with normal Fe and high functional compounds in grains (Pereira-Caro et al. 2013; Oo et al. 2025) to obtain an F_2_ population in which phenotypic segregation could be easily identified (Fig. 4a). A total of 219 F_2_ plants were grown in soil for 16–18 days after germination, and the Fe concentration of the whole shoot was measured. A total of 166 F_2_ individuals had Fe concentrations similar to those of ‘Asamurasaki,’ while 53 F_2_ individuals had Fe concentrations as high as those of the *tetsu* mutant (Fig. 4b). The chi-square test did not reject the null hypothesis of a 3:1 segregation ratio, which is consistent with the idea that the causal gene was single and recessive (Fig. 4b).

To identify the causal gene, the genotypes of F_2_ individuals were determined using a recently developed GRAS-Di system (Hosoya et al. 2019; Miki et al. 2020; Fekih et al. 2023), a method based on comprehensive amplicon sequencing. First, a comparison of the amplicons of the parents (*tetsu* and ‘Asamurasaki’) revealed 2090 amplicon markers. These DNA markers mapped widely and uniformly to all rice chromosomes, confirming that a set of good-quality markers had been obtained to identify the causal gene (Fig. 4c and Fig. S4). Next, we developed an “amplicon index” that quantified the detection or non-detection of each marker in individual F_2_ plants. The Δ (amplicon index) was calculated to identify regions where marker detection rates differed significantly between F_2_ populations with low and high Fe (Fig. S5).

This method successfully narrowed down the causative locus to the 24.3- to 30.3-Mb region of chromosome 1 (Fig. 4d). To identify all the mutation sites of *tetsu* within this causative locus, we compared the whole genome sequences of T65 and *tetsu* using high-precision next-generation sequencing analysis and identified 39 mutation sites. These were all GC/AT substitutions, a typical type of mutation known to occur after MNU mutagenesis. Among these, we found a novel nonsense mutation within the fourth exon of the *HRZ1* gene, which encodes a ubiquitin ligase predicted to function as a negative regulator of Fe-uptake/translocation-related genes (Fig. 4e, f). Other mutation sites included missense mutations in non-coding and untranslated regions, as well as putative genes with no significant reported function, none of which were potential candidates (Fig. 4e and Table S1).

### Shoot Fe Content is Increased by the *tetsu*-type *HRZ1* Mutation

**Fig. 5:**
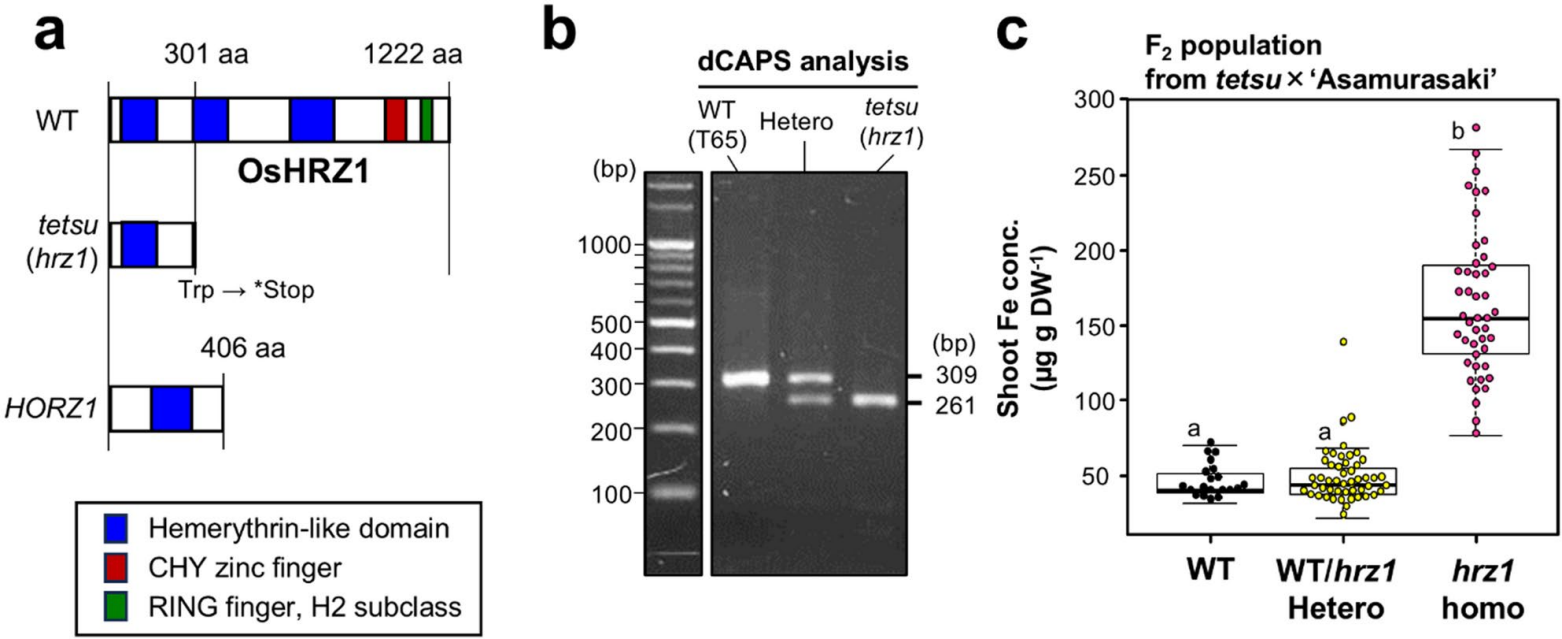
*tetsu*-type homozygous mutation in *OsHRZ1* markedly increases Fe in the shoot. **a** Comparison of the primary sequence of the OsHRZ1 protein between WT and *tetsu*(*hrz1*) mutant based on the DNA sequence. The major functional domains within the OsHRZ1 protein are also shown in the figure. **b** A dCAPS marker was developed for genotyping by detecting the presence or absence of the *tetsu*(*hrz1*) mutation. Details of this dCAPS method are described in Fig. S6. **c** Comparison of shoot Fe concentrations in different genotypes of the F_2_ population from crosses between *tetsu*(*hrz1*) and ‘Asamurasaki.’ Shoots of the 2-week-old F_2_ population rice plants grown in soil were harvested and analyzed for Fe. Means with the same letter are not significantly different at *P* < 0.05, according to Tukey’s multiple comparison test

The mutation found in *HRZ1* is presumed to express a truncated HRZ1 protein that has only the first HHE domain without the other two HHE domains and the C-terminal zinc-finger domains (Fig. 5a). The protein that can be produced by such a mutation is similar to the rice hemerythrin domain protein HORZ1(Fig. 5a), which positively regulates the Fe-deficiency response—the opposite function to the normal HRZs (Kobayashi et al. 2013; Shinkawa et al. 2025).

We developed a dCAPS marker that could efficiently detect this genetic mutation using modified forward primers and the HincII enzyme (Fig. 5b and Fig. S6). This dCAPS assay reliably detected the *HRZ1* gene as a 309 bp fragment in WT, a 261 bp fragment in the homozygous mutant, and both fragments in the heterozygous mutant (Fig. 5b). Investigating the relationship between *HRZ1* genotype and shoot Fe concentration in 114 F_2_ plants from *tetsu*×‘Asamurasaki’ discriminated by this dCAPS marker, we found that the homozygous mutant of *HRZ1* had significantly higher Fe content than did WT or heterozygous plants (Fig. 5c). This result suggests that the high Fe accumulation in the *tetsu* mutant was caused by a *tetsu*-type nonsense mutation in *HRZ1*.

### *tetsu(hrz1)* Mutant Strongly Induces an Fe-Deficiency Response at the Transcriptional Level

Previously, RNAi-knockdown lines of *HRZ2* showed increased expression of various Fe uptake- and utilization-related genes in Fe-sufficient roots, similar to Fe-deficient non-transformant rice roots (Kobayashi et al. 2013). To confirm whether the *tetsu*(*hrz1*) mutation also upregulated the expression of such genes, we performed a comparative RNA-seq analysis in WT and the *tetsu*(*hrz1*) mutant grown in normal Fe-sufficient soil (Fig. 3). Because high Fe accumulation occurred in the shoots of *tetsu*(*hrz1*) plants (Fig. 1), we investigated its expression in fully developed young leaves.

As shown in Table 1, a significant increase in the expression of various Fe deficiency–induced genes was found in the *tetsu*(*hrz1*) mutant compared with that in WT, including *IMA1*, *IMA2*, *IRO2*,* IRO3*, *NAS1*,* NAS2*,* YSL2*, *NRAMP1*, *TOM1*, *OPT7*, and *PTR* (a putative NA/DMA efflux transporter). HHE domain–containing proteins (*HRZs* and *HORZ1*) and IDEF1, which control Fe deficiency signals further upstream, were not significantly altered in the mutant. A significant, but very slight, decrease in IDEF2 expression was observed in the *tetsu*(*hrz1*) mutant. *NAS3* levels declined, consistent with previous reports that *NAS3* was repressed by Fe deficiency but induced by excess Fe (Aung et al. 2018). In addition, the expression of the ferritin genes *FER1* and *FER2*, which are known to be induced by excess Fe, was downregulated in the leaves of *tetsu*(*hrz1*). These results clearly indicate that the *tetsu*(*hrz1*) mutation enhanced the Fe-deficiency responses but suppressed the excess Fe responses in the shoot despite containing elevated Fe concentrations. No upregulation of any of the genes encoding proteins involved in the transport and regulation of other heavy metals or metal-chelating compounds (phenolics and citrate) was observed in the *tetsu*(*hrz1*) mutant (Tables 1 and 2).

**Table 1:** RNA-seq analysis of genes involved in Fe-deficiency responses, DMA synthesis/secretion, Fe transport/translocation, and Fe sequestration/storage in leaves of the wild-type (WT) and *tetsu* mutant

Transcript ID	Gene	Description	WT	tetsu (hrz1)	Fold change*	*p*-value
			transcripts per million (TPM)			
**Hemerythrin domain-containing genes**						
Os01t0689451-01	***HRZ1***	Hemerythrin motif-containing RING-& Zinc-finger protein 1	1.02 ± 0.22	0.787 ± 0.11	0.771	0.4
Os05t0551000-01	***HRZ2***	Hemerythrin motif-containing RING-& Zinc-finger protein 2	1.66 ± 0.37	1.87 ± 0.33	1.12	0.7
Os01t0861700-00	***HORZ1***	Hemerythrin motif-containing protein without RING- and Zn-finger 1	0.402 ± 0.21	0.175 ± 0.16	0.435	0.44
**Constitutively expressed Fe-related transcription factors**						
Os08t0101000-01	***IDEF1***	Iron deficiency-responsive cis-acting element binding factor 1	13.1 ± 1.81	11.1 ± 1.02	0.851	0.40
Os05t0426200-02	***IDEF2***	Iron deficiency-responsive cis-acting element binding factor 2	58.7 ± 1.40	49.6 ± 2.38	*0.845*	0.03 *
**Fe deficiency-induced Fe-related transcriptional and signaling regulators**						
Os01t0647200-01	***IMA1***	Fe-deficiency-inducible IRON MAN 1	3.24 ± 1.05	613 ± 115	**189**	0.0061 **
Os01t0647200-04	***IMA1***		12.6 ± 3.80	2170 ± 525	**173**	0.015 *
Os07t0142100-01	***IMA2***	Fe-deficiency-inducible IRON MAN 2	0.0967 ± 0.10	2340 ± 725	**> 10**,**000**	0.032 *
Os01t0952800-01	***IRO2***	Iron-related transcription factor 2, bHLH056	0.00433 ± 0.00	157 ± 21.5	**> 10**,**000**	0.0019 **
Os03t0379300-01	***IRO3***	Iron-related transcription factor 3, bHLH063	3.62 ± 0.62	22.8 ± 1.58	**6.3**	0.0004 ***
Os03t0379300-02	***IRO3***		1.31 ± 0.84	15.7 ± 2.34	**11.9**	0.0044 **
Os03t0379300-03	***IRO3***		0.112 ± 0.09	1.16 ± 0.31	**10.4**	0.031 *
**Biosynthesis of mugineic acid family phytosiderophores**						
Os03t0307300-01	***NAS1***	Nicotianamine synthase 1	0.0118 ± 0.01	2.09 ± 0.28	**177**	0.0018 **
Os03t0307200-01	***NAS2***	Nicotianamine synthase 2	0.114 ± 0.11	4.45 ± 0.86	**38.9**	0.0074 **
Os07t0689600-01	***NAS3***	Nicotianamine synthase 3	1.96 ± 0.32	0.721 ± 0.31	*0.367*	0.048 *
Os02t0306401-01	***NAAT1***	Nicotianamine amino-transferase 1	35.8 ± 7.39	15.7 ± 3.61	0.439	0.071
Os03t0237100-01	***DMAS1***	Deoxymugineic acid synthase 1	17.4 ± 1.54	24.1 ± 3.94	1.390	0.184
**Fe uptake and/or translocation**						
Os02t0649900-01	***YSL2***	Fe- and Mn-nicotianamine transporter	0.0424 ± 0.04	22 ± 4.09	**519**	0.0058 **
Os07t0258400-01	***NRAMP1***	Homologues of mammalian Nramp1, Fe transporter	0.0514 ± 0.05	36.9 ± 6.75	**718**	0.0055 **
Os07t0258400-02	***NRAMP1***		N.D.	14.9 ± 2.42	**> 10**,**000**	0.0036 **
Os07t0258400-03	***NRAMP1***		N.D.	3.69 ± 1.64	> 10,000	0.088
Os11t0134900-01	***TOM1***	Efflux transporter of mugineic acids	N.D.	0.528 ± 0.04	**> 10**,**000**	0.0002 ***
Os03t0751100-01	***OPT7***	Oligopeptide Transporter 7	9.02 ± 1.12	15.8 ± 1.14	**1.75**	0.013 *
Os03t0751100-02	***OPT7***		6.25 ± 1.32	13.1 ± 0.52	**2.09**	0.0084 **
Os01t0871500-01	***PTR***	Peptide transporter (Arabidopsis NAET-like)	44.3 ± 1.12	226 ± 25.3	**5.11**	0.002 **
Os03t0667500-01	***IRT1***	Iron-regulated transporter 1	2.23 ± 0.73	2.11 ± 0.49	0.947	0.9
Os03t0667300-01	***IRT2***	Iron-regulated transporter 2	N.D.	0.0375 ± 0.03	> 10,000	0.21
Os11t0151500-01	***ENA1***	Efflux of nicotianamine 1	0.00498 ± 0.00	0.343 ± 0.13	68.9	0.06
Os06t0695800-01	***ENA2***	Efflux of nicotianamine 2	0.630 ± 0.11	0.570 ± 0.20	0.906	0.81
Os12t0282000-01	***MIR***	Mitochondrial iron-regulated gene	N.D.	0.288 ± 0.18	> 10,000	0.18
Os04t0463400-01	***VIT1***	Vacuolar Iron Transporter 1	15.5 ± 0.75	18.5 ± 1.44	1.19	0.14
Os09t0396900-02	***VIT2***	Vacuolar Iron Transporter 2	6.41 ± 2.10	0.783 ± 0.19	0.122	0.056
Os11t0106700-01	***FER1***	Ferritin 1	25.9 ± 2.58	15.1 ± 0.71	*0.584*	0.016 *
Os11t0106700-02	***FER1***		244 ± 27.8	131 ± 19.3	*0.536*	0.029 *
Os12t0106000-01	***FER2***	Ferritin 2	296 ± 39.4	151 ± 21.4	*0.512*	0.032 *

* Fold-change indicates the expression ratio of *tetsu*/WT. Bold numbers indicate genes in the *tetsu* mutant that were significantly upregulated, whereas underlined numbers indicate genes that were significantly downregulated. WT and *tetsu* homozygotes were isolated from the F_2_ population obtained after a single backcross to T65 for the *tetsu* mutant, with three biological replicates (*n* = 3). Plants were grown under normal soil conditions for 60 days at 26 °C in a greenhouse. Young leaves (6th leaf) were harvested at 2:30 p.m. Data are expressed as transcripts per million. Significant differences (* *P* < 0.05, ** *P* < 0.01, *** *P* < 0.001) between the WT and *tetsu* mutants were tested using the Student’s t-test

**Table 2:** RNA-seq analysis of transporters related to heavy metals in leaves of the wild-type (WT) and *tetsu* mutant

Transcript ID	Gene	Description	WT	tetsu (hrz1)	Fold change*	*p*-value
			transcripts per million (TPM)			
**Other transporters related to translocation of Fe or other metals**						
Os07t0232800-01	***ZIP8***	Zinc transporter 8	96.7 ± 14.4	32.9 ± 10.4	*0.340*	0.023 *
Os07t0232800-02	***ZIP8***	Zinc transporter 8	4.69 ± 0.41	2.01 ± 0.82	*0.429*	0.043 *
Os03t0571900-01	***PEZ1***	Phenolics efflux zero 1	6.48 ± 0.54	4.45 ± 1.46	0.686	0.26
Os03t0572900-01	***PEZ2***	Phenolics efflux zero 2	34 ± 0.90	30.2 ± 5.12	0.888	0.51
Os12t0133100-02	***VMT***	Vacuolar mugineic acid transporter (ZIFL12)	16.5 ± 2.50	16.3 ± 1.22	0.986	0.937
Os03t0216700-01	***FRDL1***	Citrate transporter (Fe-citrate transport)	1.86 ± 0.29	1.72 ± 0.72	0.927	0.870
Os10t0206800-01	***FRDL2***	Citrate transporter (Al-induced)	14.3 ± 0.67	10.43 ± 1.88	0.731	0.128
Os10t0206800-02	***FRDL2***	Citrate transporter (Al-induced)	6.57 ± 0.58	4.99 ± 0.80	0.759	0.183
Os02t0833100-01	***FRDL3***	Citrate transporter, putative	1.09 ± 0.09	1.56 ± 0.28	1.43	0.185
Os01t0919100-01	***FRDL4***	Citrate transporter (Al-induced)	N.D.	N.D.	-	-
Os02t0650300-01	***YSL15***	Fe- and Mn-nicotianamine transporter	N.D.	N.D.	-	-
Os06t0560000-02	***FPN1***	Ferroportin, intracellular Ni and Co transporter (IREG1)	2.55 ± 0.22	2.99 ± 0.45	1.17	0.424
Os07t0257200-01	***NRAMP5***	Low cadmium accumulation 1	0.580 ± 0.31	0.613 ± 0.31	1.06	0.940
Os07t0232900-01	***HMA3***	Heavy metal ATPase 3	0.801 ± 0.25	0.973 ± 0.24	1.22	0.640

* Fold-change indicates the expression ratio of *tetsu*/WT. Underlined numbers indicate genes that were significantly downregulated in the *tetsu* mutant. The expression values were obtained from the same dataset, as shown in Table 1

### Attempt to Increase Fe Content in Rice Grain while Simultaneously Increasing Antioxidant Components

**Fig. 6:**
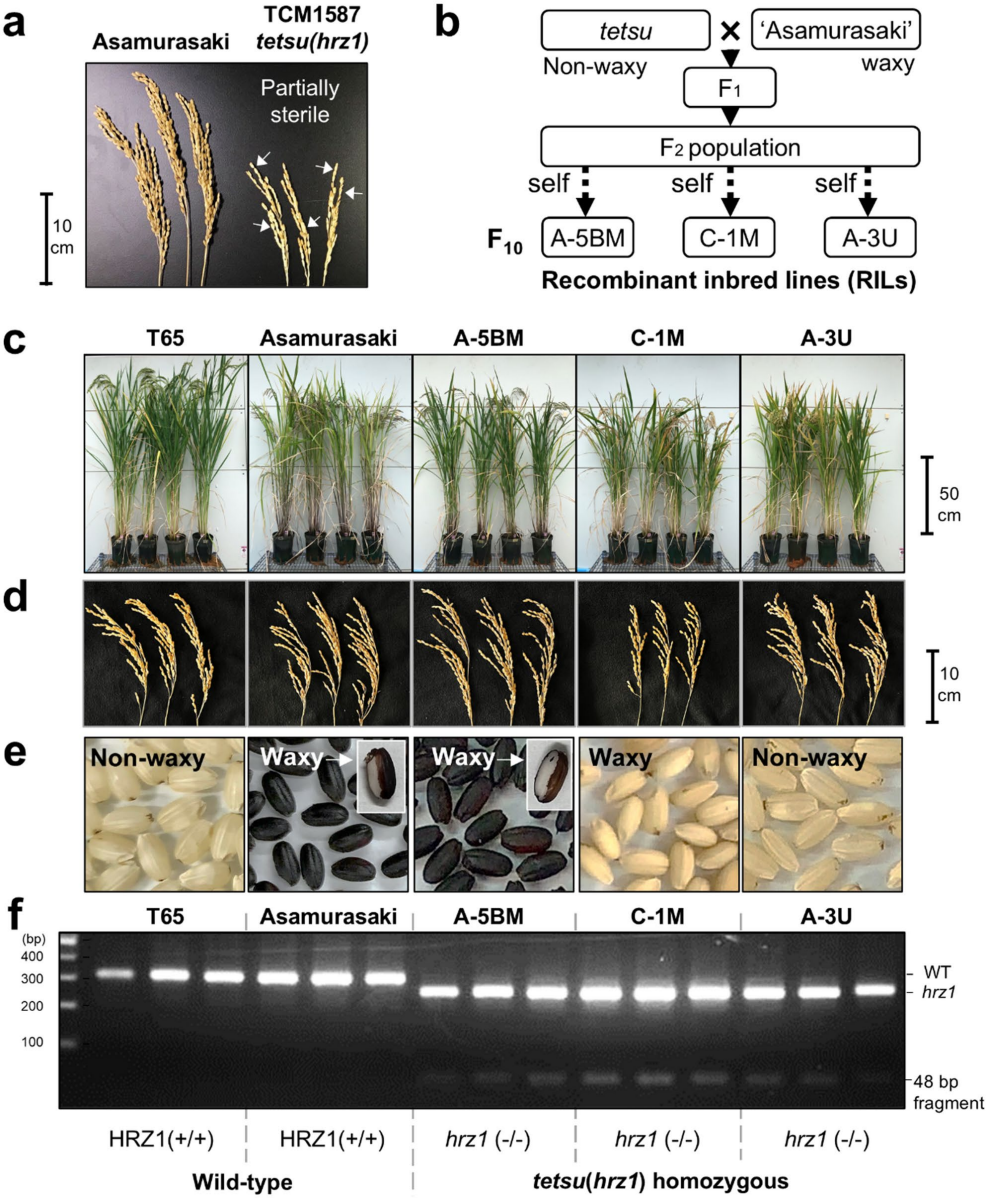
RILs with the*tetsu*(*hrz1)*mutation show sufficient fertility and well-developed seed formation. **a** TCM1587, which is the original ancestral lineage of the *tetsu*(*hrz1*) mutant, exhibited low fertility and small panicles. The typical undeveloped seeds are indicated by the arrow. **b** Schematic diagram of the breeding steps to obtain the RILs from the *tetsu*(*hrz1*) and “Asamurasaki.” **c**–**d** Image of plants at heading period and the panicle taken from three individual plants. RILs showing the normal appearances. **e** Characteristics of brown rice (anthocyanin coloration and waxy properties). **f** dCAPS analysis showing three RILs have a homozygous *tetsu*(*hrz1*) mutation

Sterility occasionally occurred in the original TCM1587 mutant line used for selecting the *tetsu*(*hrz1*) mutant, independent of high Fe accumulation (Fig. 6a). This is due to the vast number of genomic mutations that occur randomly in the MNU mutagen of the original *tetsu*(*hrz1*) genome. Therefore, we further screened lines with improved defective traits from the progeny of the F_2_ population (*tetsu* × ‘Asamurasaki’).

After 10 generations of repeated selfing, we obtained pure recombinant inbred lines (RILs) (Fig. 6b). Among the RILs, A-5BM, C-1 M, and A-3U showed normal growth and fertility comparable to those of T65 and ‘Asamurasaki’ (Fig. 6c, d). Because the parent cultivar ‘Asamurasaki’ is a glutinous (waxy) black–purple rice that accumulates high concentrations of many functional components and antioxidants in its grains (Pereira-Caro et al. 2013), the selected RILs inherited these traits in various combinations, i.e., A-5BM, which is black–purple rice and glutinous (waxy), C-1 M, which is white rice and glutinous (waxy), and A-3U, which is white rice that is not glutinous (non-waxy) (Fig. 6e). Using the dCAPS assay, we confirmed that the three RILs harbored a *tetsu*(*hrz1*) mutation (Fig. 6f).

**Fig. 7:**
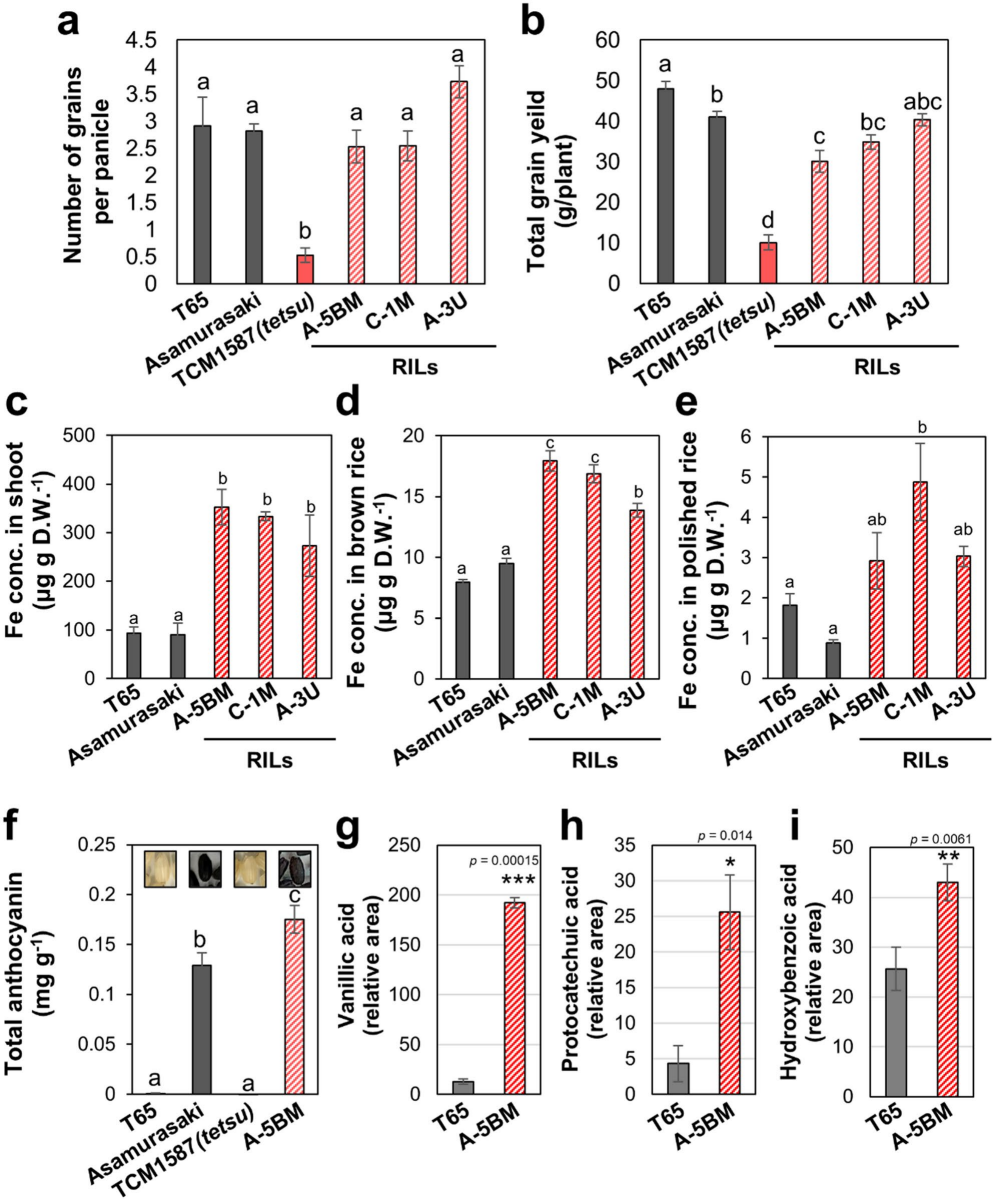
RILs with the *tetsu*(*hrz1*) mutation exhibit high-Fe accumulating characteristics with normal grain yield. RILs improved the grain number per panicle (**a**) and total grain yield (**b**) compared with those in TCM1587, the original ancestral lineage of the *tetsu*(*hrz1*) mutant. Fe concentrations in shoots (**c**), brown rice (**d**), and polished rice (**e**) were significantly increased in RILs with *tetsu* (*hrz1*) mutation compared with their parental cultivars, T65 and ‘Asamurasaki.’ The total shoots and brown rice harvested after maturity were analyzed for the same rice used in Fig. 6. Plants were grown on soil until fully ripe in a controlled greenhouse under natural light and supplemental light with a light intensity of more than 400 µmol m^− 2^ s^− 1^ during the day, with a 14 h light (28 °C)/10 h dark (25 °C) cycle. Of the RILs, A-5BM accumulated higher concentrations of anthocyanins and phenolic compounds in brown rice than T65 (**f**–**i**). Each column represents the mean ± standard error. Means with the same letter are not significantly different at *P* < 0.05, according to Tukey’s multiple comparison test or with asterisks indicating significant differences (* *P* < 0.05, ** *P* < 0.01, *** *P* < 0.001) between T65 and *tetsu* using Student’s *t*-test (*n* = 3)

Under normal soil conditions, the three RILs exhibited grain yield levels comparable to those of the WT cultivars T65 and ‘Asamurasaki,’ indicating that the reduced yield observed in the original *tetsu*(*hrz1*) mutant was largely restored by crossing. For example, the number of grains per panicle was significantly lower in the original *tetsu*(*hrz1*) mutant, but was comparable among the three RILs and the WT cultivars (Fig. 7a). Similarly, the total grain weight was significantly improved in the three RILs compared with that in the *tetsu*(*hrz1*) mutant (Fig. 7b). Because yield-related traits are often influenced by growth duration, we compared the developmental timing of the parental cultivars and these RILs carrying the *tetsu*(*hrz1*) mutation. The RILs exhibited intermediate heading dates and total cultivation days between the early-maturing cultivar ‘Asamurasaki’ and the late-maturing cultivar T65 (Table S2). Taken together, these results indicate that the improved yield observed in these RILs is not attributable to differences in growth duration. Shoot Fe concentrations in the three RILs remained significantly increased by approximately 3-fold compared with those in WT cultivars (T65 and ‘Asamurasaki’) (Fig. 7c). Fe concentrations in brown rice were also up to 2-fold higher in these RILs than in WT cultivars (Figs. 7d). Fe concentrations in polished rice also showed an increasing trend, averaging 3- to 5-fold higher compared with those in ‘Asamurasaki’ and 1.6- to 2.7-fold higher than those in T65 (Fig. 7e). Thus, RILs that regained normal reproductive growth through crossing with ‘Asamurasaki’ maintained stable, high Fe contents across generations, demonstrating that the *tetsu*(*hrz1*) mutation itself is not a harmful mutation that reduces yield potential. Among these RILs, A-5BM had significantly more antioxidant components, such as anthocyanins and various phenolic acids, which are abundant in ‘Asamurasaki’ (Fig. 7f–i). These results indicate that the identified novel *HRZ1* mutation is a valuable target for engineering non-transgenic Fe-biofortified rice cultivars with various beneficial traits.

## Discussion

We identified a new nonsense mutation in *HRZ1* (Figs. 4 and 5) in the Fe-accumulating mutant *tetsu*. Notably, RILs with the *tetsu(hrz1)* mutation (Figs. 6 and 7) or the backcross line of the *tetsu*(*hrz1*) mutant (Fig. S3) did not show abnormalities in growth or fertility, with a high Fe content in both the leaves and seeds. This contrasts with the previously reported *hrz1-1* mutant (Kobayashi et al. 2013) or *hrz1-2* genome editing line (Zhang et al. 2017), both of which are reportedly sterile and/or have poor growth. Because of these undesirable characteristics, achieving Fe biofortification using the previously reported *HRZ* knockout/knockdown lines has been challenging. More recently, Shinkawa et al. (2025) created rice plants with mutated *HRZ1* using a transgenic strategy and CRISPR/Cas9 genome editing techniques. These transformants/mutants targeting *HRZ1* exhibited Fe deficiency tolerance and increased Fe content in brown rice, similar to the *tetsu*(*hrz1*) mutant. However, the shoot Fe content, yield potential, and sterility of these transformants/mutants have not been reported in detail.

Among the *hrz1* mutants reported to date, *hrz1-1* showed little or no change in shoot Fe compared with that in WT (Kobayashi et al. 2013). This contradicts the *tetsu(hrz1)* mutant, which accumulated more than 3-fold as much Fe in the shoots as the WT (Figs. 1c, 5c and 7a). The factor that caused differences between the previously reported *hrz1* mutants and the current *tetsu(hrz1)* mutant is likely the difference in the mutation site. The *hrz1-1* mutant line had a DNA insertion upstream of the 5’-UTR, causing HRZ1 expression to be moderately suppressed. In contrast, the *tetsu(hrz1)* mutant has a nonsense mutation caused by a single-base substitution at the beginning of the second HHE domain, which translates into a truncated HRZ1 with only one complete HHE domain. The shortened structure of the HRZ1 protein was similar to that of another HHE-containing protein, HORZ1 (Fig. 5a), which functions as a positive regulator of Fe-deficiency responses. Thus, it is possible that the mutated HRZ1 protein accumulates in the *tetsu*(*hrz1*) mutant. This truncated protein may function as a positive regulator of Fe-deficiency responses. This hypothesis is supported by the findings of Shinkawa et al. (2025), which suggest that overexpression of GFP-HRZ1ΔRZ constructs, comprising only two hemerythrin domains of HRZ1 without the other domains, enhances Fe deficiency responses in a dominant-negative manner due to the mutated shortened HRZ1 proteins. Further research is required to confirm our hypothesis regarding the *tetsu*(*hrz1*) mutation.

Among the genes upregulated in the *tetsu(hrz1)* mutant (Table 1), *IMA1*, *IMA*2, *IRO2*, and *IRO3* were significantly upregulated at the transcriptional level in shoots. In addition, Fe deficiency–inducible genes such as *YSL2*, *TOM1*, *ENA1*, *NRAMP1*, and *NAS1/2* were strongly upregulated in the shoots of the *tetsu(hrz1)* mutant. Earlier reports have shown that suppressing *HRZ1/2* expression using RNAi causes high expression of these Fe-deficiency response genes (Kobayashi et al. 2014). Thus, the ability of the *tetsu(hrz1)* mutant to accumulate high levels of Fe in the shoots is assumed to be the reason for the enhanced Fe-deficiency responses. Furthermore, the *tetsu(hrz1)* mutant exhibited a 5-fold increase in the putative NA/DMA efflux transporter *PTR* (Os01g0871500) (Nozoye et al. 2011) and a 2-fold increase in the expression of *OPT7* (Table 1). The gene with high homology to *PTR* is *NAET*1/2 in Arabidopsis, which is responsible for Fe source-to-sink transport (Chao et al. 2021). OPT7 is a transporter responsible for the xylem unloading of Fe^2+^ and the preferential distribution of Fe in developing tissues (Bashir et al. 2015; Yamaji et al. 2024). Thus, the increased expression of *PTR* and *OPT7* in the *tetsu(hrz1)* mutant could contribute to the marked increase in Fe concentration in the grains (Fig. 1).

In addition to Fe, the *tetsu(hrz1)* mutant line accumulated high Mn and Ni levels in the shoots (Fig. 1 and Fig. S1) and Zn and Cu in the grains (Figs. 4 and 5). This can be explained by the fact that DMA and NA not only bind to Fe but also have an affinity for Mn^2+^, Ni^2+^, Cu^2+^, and Zn^2+^, thereby facilitating the translocation and transport of these essential transition metals in shoots (Murakami et al. 1989; Mari et al. 2006; Curie et al. 2009). In contrast, the contents of non-essential Cd, Pb, and Co were not increased in the shoots of the *tetsu(hrz1)* mutant (Fig. S1). Although recent reports indicate that the overexpression of NAS in yeasts or Arabidopsis enhances Cd mobility (Hollmann et al. 2025), we did not obtain any data indicating that the *hrz1* mutation enhances Cd uptake. This is likely because the primary Cd uptake pathway in rice is OsNRAMP5, which transports Cd²⁺, the predominant chemical form in aerobic paddy fields (Ishikawa et al. 2012; Sasaki et al. 2012). Furthermore, we confirmed the absence of increased expression of any transporters associated with heavy metal transport in the *tetsu(hrz1)* mutant (Table 2), including *OsZIP*s (Zn and Cd transporters), *OsNramp5* (Cd, Pb, and Mn transporters), *OsHMA3* (vacuolar Cd transporter), and *FPN1* (Ni and Co transporters; Kaur et al. 2021; Kan et al. 2022). These results revealed that the *tetsu*(*hrz1*) mutation could serve as a beneficial target gene site for improvement in Fe biofortification, simultaneously increasing the Zn and Cu content in grains while preventing the absorption of harmful elements.

When developing Fe-biofortified crops using *hrz*/*bts* mutations, considering the risk of Fe toxicity in plants is essential. Unlike rice, most other plant species are sensitive to excess Fe. Indeed, severe Fe toxicity with defective embryo development and leaf necrosis could occur because of increased amounts of Fe in the shoots of Arabidopsis *bts* mutants and a pea *HRZs/BTS* ortholog mutant *dgl* (*degenerate leaves*) grown under normal soil conditions (Welch and Larue 1990; Selote et al. 2015; Harrington et al. 2024). Thus, for crops that are not tolerant to Fe toxicity, it may be challenging to confer a trait that significantly increases Fe content in shoots, such as the *tetsu*(*hrz1*) mutant. Previous reports have indicated that *hrz1* knockdown rice is more prone to Fe toxicity when grown under Fe-excess conditions (Aung et al. 2018; Kobayashi et al. 2019). In the present study, bronzing, an indicator of excess Fe, occasionally appeared on the leaf tips during later growth stages. However, no adverse effects on yield due to excess Fe were observed in RILs carrying the *tetsu*(*hrz1*) mutation under the soil conditions used (Fig. 7). Notably, we have repeatedly conducted seed multiplication and agronomic cultivation of these RILs across multiple field sites, and to date we have not observed sterility attributable to Fe toxicity. Under these field conditions, grain yields of RILs were generally in the range of approximately 300–400 kg per 10 a, which is comparable to those of nutritionally rich rice cultivars such as ‘Asamurasaki’. Thus, we have obtained no clear evidence that excess Fe accumulation in these RILs causes a marked reduction in yield under practical field conditions. Nevertheless, further improvement through crossing with high-yielding cultivars or Fe-toxicity-tolerant varieties will be an important future strategy to ensure stable productivity even under potentially Fe-excess environments. In this context, recently reported rice lines possessing excess Fe tolerance traits (Rosdianti et al. 2025) may be a promising option for breeding materials for crossing with these RILs to enhance yield stability.

Although various attempts have been made to develop Fe-biofortified rice using the transgenic strategy, its widespread commercialization is limited by the difficulty in obtaining approval or acceptance of such genetically modified plants in various countries. In this context, mutation breeding with a DNA marker for the *tetsu*-type mutation is an effective strategy to facilitate the practical application of Fe biofortification in non-genetically modified rice. Here, we succeeded in developing rice with increased Fe and other nutritional, polyphenol, and phenolic components (Fig. 7). The RILs generated in this study can be used in regular edible rice or processed rice products. It should be noted that the phenotypic stability and efficiency of Fe accumulation conferred by the *tetsu*(*hrz1*) mutation may vary depending on the genetic background. Although the RILs analyzed in this study were derived from japonica backgrounds (T65 and ‘Asamurasaki’), the applicability of this mutation to diverse rice genetic backgrounds including Indica rice and African rice subspecies remains to be determined. Regarding this, we have recently demonstrated that shoot Fe deficiency responses and their tolerance levels vary considerably among rice subspecies (Saito et al., in press). Therefore, future introduction and evaluation of the *tetsu*(*hrz1*) mutation in a wide range of rice cultivars and subspecies will be important to assess its broader utility and potential for widespread adoption in rice breeding.

In summary, we successfully established Fe-biofortified rice lines that exhibited normal growth, fertility, and various brown rice traits by breeding a *tetsu*(*hrz1*) mutant. These results confirmed that the identified novel *HRZ1* mutation is a valuable target for engineering non-transgenic Fe-biofortified rice cultivars. Currently, we are conducting safety tests in animals to confirm their efficacy and anticipate that these RILs will become widely available.

## Materials and Methods

### Plant Materials and Growth Conditions

T65 (accession no. T0504) seeds were provided by the National Institute of Genetics (Japan). The T65 MNU-induced rice mutants (TCM mutants) were generated by T.Kumamaru (Kyushu University) and provided to T.F. or A.S. through the National BioResource Project (MEXT, Japan). ‘Asamurasaki’ seeds were purchased from a Japanese seed company (Noguchi Seeds Co., Saitama, Japan). For the primary screening of the TCM mutants, 128-cell plug trays (Takii Seed Co., Kyoto, Japan) were used for cultivation with commercial soil at pH 5.0, N: P:K = 0.8:2.0:1.0 (Kumiai Chemical Industry Co., Tokyo, Japan). The mineral content of the soil was determined as described previously (Uraguchi et al. 2011). The seeds were disinfected by soaking in 60 °C water for 10 min, followed by watering for 3 days to promote germination. For each seedling tray, eight plants of the T65 and 90 lines of TCM mutants were sown, totaling 2887 TCM lines. The plants were cultivated for 21 days under natural light in a greenhouse. For the cultivation of *tetsu* mutant selected from the seed pool of TCM1587 was grown on soil in an air-conditioned greenhouse (26 ± 3 °C). For yield surveys of the *tetsu(hrz1)* mutant and RIL lines, plants were grown in soil in a controlled greenhouse under natural light and supplemental light with a light intensity of more than 400 µmol m^− 2^ s^− 1^ during the day, with a 14 h light (28 °C)/10 h dark (25 °C) cycle. For the alkaline soil experiments, young seedlings were pre-cultured in soil (pH 5.0) for 38 days and then transferred to soil with pH 5.0 or 8.7.

### Xylem sap collection for determining metal concentrations

Xylem sap collection was performed according to previous reports (Uraguchi et al. 2011; Yamamura et al. 2024) with minor modifications. Briefly, the aboveground portion was cut at a point 3 cm above the ground using a razor blade. A 1-mL chip filled with small pieces of quartz wool was placed over the cut surface, and the exuding xylem sap was absorbed into the quartz wool over a 3-hour period. The 1-mL chip containing quartz wool was inverted and placed into a 2-mL tube, and the xylem sap was collected by centrifugation at 3,500 rpm for 2 min. The collected xylem sap was weighed, stored at − 80 °C until analysis.

### Analysis of Metal Concentration in Rice Plants

In the metal analysis of shoots during primary screening, up to 100 mg of homogenized dry rice leaves was added to a Teflon-coated centrifuge tube, and 3 mL of a mixture of concentrated nitric acid and hydrogen peroxide (3:1) was added. The sample was placed in a heat block and heated at 80 °C for 1 h and 100 °C for 20 min, then dissolved by a vortex mixer. The reaction was continued at 150 °C for 4 h, with the acid mixture added as needed to complete the reaction. After drying, the sample was resuspended in 0.08 N nitric acid containing 2 ppb indium (In) as an internal standard. For secondary screening and subsequent analyses, 50 mg or less of dried rice leaf powder or 5–10 grains of brown rice were dissolved in 5 mL of concentrated nitric acid. The collected xylem sap was diluted with a fixed volume of 1 N nitric acid. The supernatants of the inorganic element extraction solutions were analyzed using inductively coupled plasma mass spectrometry (ICP-MS, model SPQ 9700; SII Nano Technology, currently Hitachi High-Tech Science, Inc.) for the 1st screening and a furnace atomic absorption spectrophotometer (AA-6300 with GFA-EX7i, Shimadzu, Tokyo, Japan) for the 2nd screening as described (Saito et al. 2021).

### Iron Staining of Rice Seeds Using Perls’ Staining

Perls’ staining was performed by adding 1 mL of Perls’ staining solution (4% v/v HCl and 4% w/v K-ferrocyanide) (Roschzttardtz et al. 2009) to 7–8 cut brown rice grains, followed by staining for 90 min. After washing with ultrapure water, images were captured using a stereomicroscope. To prepare longitudinal brown rice sections, one side of the brown rice was adhered to the surface of the cap of a 2 mL screw tube using instant adhesive (Aron α, Quick-Setting Multi-Purpose Extra Type, Konishi Co., Ltd., Osaka, Japan). The 2-mL tube cap, with the rice-adhered side facing upward, was then fixed to the sample tray of a microtome (DTK-1000, DOSAKA EM Co., Ltd., Kyoto, Japan) using the same instant adhesive. A razor blade was aligned with the center of the rice germ, and the sample was cut in half using a microtome set to a vibration frequency of 10 Hz (maximum value) and a cutting speed of 2–3 mm/s. The side without the adhesive was used for Perls’ staining.

### Measurement of SPAD Values in Leaves

The chlorophyll concentration indices of the leaves were measured using a mobile device (SPAD-50-Plus; KONICA MINOLTA JAPAN, Inc., Tokyo, Japan), and the average of three central areas of the latest expanded leaves was measured for each plant.

### Soil Analysis

Soil pH was measured by placing 10.0 g of air-dried soil in a 100-mL glass container, adding 100 mL of pure water, and shaking at 25 °C and 135 rpm for 24 h until the pH of the alkaline soil solution reached equilibrium. The pH of the supernatant was then measured. Soil-soluble Fe content was determined according to a well-established method (Lindsay and Norvell 1978). Briefly, a 20-mL diethylenetriamine-N, N,N’,N’’,N’’-pentaacetic acid (DTPA) extraction solution (5 mM DTPA, 0.1 M triethanolamine hydrochloride, 10 mM calcium chloride, adjusted to pH 7.3 with NaOH) was added to 10.0 g of air-dried soil. The mixture was shaken at 25 °C and 135 rpm for 2 h. The supernatant was filtered through No. 2 filter paper (Advantech Co., Ltd., Tokyo, Japan), and nitric acid was added to achieve a final concentration of 1%. The DTPA-extracted Fe was then measured using furnace atomic absorption spectroscopy (AA-6300 with GFA-EX7i, Shimadzu, Tokyo, Japan).

### Data Collection Procedures for GRAS-Di

A total of 97 individuals from the F_2_ population and their parents were used for GRAS-Di analysis (Toyota Motor Corporation, Tokyo, Japan). First, germinated seeds were grown in soil for 16–18 days, and then the shoots were cut with a razor blade. The Fe content of the shoots was analyzed in advance. After approximately 40 days, new shoots regenerated from the remaining plant stems. Based on the Fe content values, 54 high-Fe F_2_ individuals and 50 low-Fe F_2_ individuals, or 3 individuals from each parent, were selected. Healthy and most recently expanded leaves were collected, washed, and immediately frozen in liquid nitrogen. The leaves were then ground to a fine powder using a mortar and pestle under liquid nitrogen, and genomic DNA was extracted using a DNeasy Plant Mini Kit (69104; QIAGEN). GRAS-Di libraries were constructed using a NovaSeq 6000 S4 reagent kit. Libraries were sequenced using an Illumina NovaSeq 6000 (Sequence Mode: 2 × 150; Flow Cell Type: S4; Illumina, San Diego, CA, USA). Genotyping was conducted using 38,950 dominant single-dose markers generated using GRAS-Di software ver. 1.0.5. (TOYOTA, Aichi, Japan). Sequence reads derived from sequence adapters and those with low sequence quality were excluded from data analysis. Among the resulting 14,757 amplicons, those showing clear amplification differences between the parents were selected. As a result, 1,068 amplicons positive only in the *tetsu(hrz1)* mutant were designated as C1, and 1,022 amplicons positive only in ‘Asamurasaki’ were designated as C2, yielding a total of 2,090 dominant GRAS-Di markers. The markers obtained were used to determine candidate positions in the reference genome sequence.

### Whole Genome Sequencing Analysis of T65 and *tetsu(hrz1)* Mutant

Five WT (T65) and eight *tetsu(hrz1)* mutant plants exhibiting a high-Fe phenotype were cultivated for 1 month after sowing. Genomic DNA was extracted from young leaves using the DNeasy Plant Mini Kit (69104; QIAGEN). Genomic DNA from each individual was mixed in a single tube to ensure equal amounts, resulting in bulk DNA samples for T65 and the *tetsu(hrz1)* mutants. Bulk DNA samples were analyzed using a Genomic DNA ScreenTape Assay (Agilent Technologies, Santa Clara, CA, USA) to determine the DNA Integrity Number (DIN), which was approximately 7, confirming the high integrity of genomic DNA. Genome shotgun analysis was performed using HiSeq X, with a read length of 2 × 150. Data analysis involved cleaning the reads using Trimmomatic software (ver. 0.39) and mapping them to the reference sequence using BWA (ver. 0.7.17). The reference sequence used was “*Oryza sativa*, IRGSP-1.0.” Both samples contained more than 200 Mb of reads, with 99.8% of the reads mapped. Additionally, over 90% of mapped reads covered the reference sequence at 50× depth, yielding high-precision sequence information. Picard tools (ver. 1.111) was used to remove the duplicate reads. Finally, we listed the bases that did not match T65 and the *tetsu(hrz1)* mutant (Table S1). The identified *tetsu(hrz1)* mutation sites were confirmed using Sanger sequencing.

### dCAPS Method to Detect the Mutation in *HRZ1* Gene

To detect *tetsu*-type mutations in *HRZ1*, we created primers based on dCAPS Finder 2.0 (http://helix.wustl.edu/dcaps/dcaps.html) (Neff et al. 2002), setting the value for mismatches to 1. Among the lists of candidate primers, we selected the following primer sets that can cleave the objective PCR products with HincII: 5’-CTA TTG ATG GTC AGG TTG AAA GGC ATC CCA TAG ATG AGA TTC TGT GTT G-3’ (49 bp) for the forward primer and 5’-AAC CTG AAT ACA CTA AGA GAA AGG T-3’ (25 bp) for the reverse primer. Genomic DNA for PCR was extracted based on conventional methods (Edwards et al. 1991). Scissored young rice leaves less than 1 cm in length were crushed with two stainless steel beads in a 2-mL tube, and the supernatant was collected and purified using the isopropanol precipitation method. More detailed information regarding the dCAPS method is provided in Fig. S6.

### RNA-Seq Analysis

From the F_3_ population that was backcrossed T65 with the *tetsu(hrz1)* mutant, homozygous WT (*HRZ*+/+) and *tetsu* (*hrz1-/-*) plants were selected. Leaves from two individuals were combined into one sample, and six individuals (*n* = 3) were analyzed. The plants were grown in a greenhouse at 26 °C under natural light for 60 days after sowing in culture soil (Bonsol No. 1; Sumitomo Chemical, Tokyo, Japan), and the youngest maximum expanded leaves (sixth leaf) of the main culms were used for analysis. Approximately 100 mg of the frozen leaf powder that had been ground in a mortar and pestle was transferred to a 2-mL tube (TM-626; TOMY MEDICO. Ltd., Tokyo, Japan) containing the RNA extraction solution of an RNeasy Plant Mini Kit (74904; QIAGEN), and crushed using two stainless steel beads (SUB-50, 4.8 mm φ; TOMY MEDICO. Ltd.) in a bead-crushing device (Micro Smash TM MS-100; TOMY MEDICO. Ltd.) at 4,000 rpm for 100 s. Genomic DNA was removed through DNase treatment, according to the standard protocol for the RNeasy Plant Mini Kit. cDNA was prepared by targeting RNA with a poly A tail using the SMART-Seq v4 Ultra Low Input Kit (#634888; Clontech Laboratories, Inc., Mountain View, CA, USA). A Nexera XT DNA Library Prep kit (Illumina) was used to create a cDNA library, and at least 80 million reads were sequenced per sample using NovaSeq (Illumina). Data analysis was performed using the DRAGEN Bio-IT Platform (version 3.7.5; Illumina). The read sequences obtained from the sequencing analysis were mapped to the reference genome sequence (*Oryza sativa* IRGSP-1.0), and approximately 99% of all reads were identified. The expression levels of the genes and transcripts were calculated based on the positional information obtained from the mapping and gene definition files.

### Measurement of Total Anthocyanin in Rice Grains

Anthocyanin content was measured according to Watanabe et al. (2014). Approximately 100 mg of brown rice was mixed with 1 mL of anthocyanin extraction solvent (methanol: water: trifluoroacetic acid = 40:60:0.5), and the mixture was incubated at 37 °C overnight. The mixture was then ground four times using a bead-crushing device (Micro Smash TM MS-100, TOMY MEDICO. Ltd.) with three zirconia beads (φ3 mm) at 4,000 rpm for 120 s, with ice cooling applied each time. Centrifugation was performed at 15,000 × *g* for 10 min at 4 °C, and the supernatant was collected. The supernatant was diluted 5-fold with the extraction solvent, centrifuged at 15,000 × *g* for 5 min at room temperature to remove insoluble residues, and the absorbance was measured at 525 nm. Total anthocyanin content was calculated based on the molar extinction coefficient of cyanidin-3-glucoside (Singh et al. 2022).

### Measurement of Phenolic Acids in Rice Grains

A total of 600 µL methanol containing 50 µM internal standards (L-methionine sulfone, 2-morpholinoethanesulfonic acid, and ^13^C_6_-glucose) was added to 45–50 mg of brown rice powder and mixed using a bead-crushing device under cooling conditions (1,500 rpm, 120 s × 2 times). Thereafter, 600 µL of Milli-Q water was added, stirred, and centrifuged (2,300 × *g*, 4 °C, 5 min). The supernatant was transferred to an ultrafiltration tube (UltraFree-MC-PLHCC, 5 kDa ultrafiltration membrane, Human Metabolome Technologies, Inc. Yamagata, Japan), and centrifuged (9,100 × *g*, 4 °C, 120 min). Subsequently, the filtrate was dried, dissolved in an aliquot of Milli-Q water, and used for measurements. The sample was analyzed using an Agilent CE-TOFMS system (Agilent Technologies) with a fused silica capillary (i.d. 50 μm × 80 cm) in anion mode. Peaks detected using CE-TOFMS were automatically extracted using the automatic integration software MasterHands ver.2.19.0.2. The relative area values were calculated by dividing the area of the target peak by the product of the area of the internal standard and sample amount. Based on the m/z and MT values, all substances registered in the metabolite library were compared to identify phenolic compounds. The acceptable error for the search was set to ± 0.5 min for MT and ± 10 ppm for m/z. For the obtained peaks, the relative area value ratios for each group were calculated and *t*-tests were performed.

### Statistical Analysis

Comparisons between two groups were performed using Student’s t-test, and multiple group comparisons were analyzed by one-way ANOVA followed by Tukey’s test. All statistical analyses were conducted using Microsoft Excel and Statistics Kingdom (2017): ANOVA Calculator (https://www.statskingdom.com/180Anova1way.html).

## Supplementary Information

Below is the link to the electronic supplementary material.

Supplementary Material 1

## Data Availability

The datasets generated and/or analyzed during the current study are available from the corresponding author on reasonable request.

## Abbreviations

MNU: N-methyl-N-nitrosourea
T65: Taichung-65
WT: Wild-type
GRAS-Di: Genotyping by Random Amplicon Sequencing-Direct
HRZ1/2: Hemerythrin (or Hemerythrin) motif-containing Ring Zinc-finger protein 1/2
RIL: Recombinant Inbred Line
IRT1/2: Iron-Regulated Transporter ½
DMA: 2’-deoxymugineic acid
NAS: Nicotianamine Synthase
NA: Nicotianamine
NAAT: Nicotianamine Aminotransferase
DMAS: Deoxymugineic Acid Synthase
MA: Mugineic acid
TOM1: Transporter of Mugineic acid family phytosiderophores 1
YS1: Yellow Stripe 1 transporter
YSL: YS1-Like transporter
IDS2/3: Iron Deficiency Specific clone no. 2/3 (DMA dioxygenases)
IDEF1/2: Iron-Deficiency-responsive Elements-binding transcription Factor ½
OsFRDL1: Ferric Reductase Defective Like 1
PEZ1/2: Phenolics Efflux Transporter ½
ENA: Efflux transporter of NA
VMT: Vacuolar Mugineic acid Transporter
VIT1/2: Vacuolar Iron Transporter ½
FER: Fe-storage protein ferritin
MIT: Mitochondrial Iron Transporter
OsIRO2/3: Iron-regulated transcription factor 2/3
bHLH: Basic Helix-Loop-Helix
PYE: POPYE, BTS, BRUTUS
HHE: Histidine histidine, and glutamic acid-containing domain
HORZ1: Hemerythrin motif-containing protein without RING- and Zn-finger 1
IMA1/2: IRON MAN/ FE uptake inducing Peptide
DTPA: Diethylenetriamine-N, N,N’,N’’,N’’-pentaacetic acid
