# Supplementary material for: Establishment of a Non-transgenic Iron-Biofortified Rice Line Using a Novel HRZ1 Mutation
Source: Rice (N Y). 2026 Mar 13;19:24. doi: 10.1186/s12284-026-00897-6 (PMC13046958; doi:10.1186/s12284-026-00897-6)
Supplement: Supplementary file 1 — Supplementary Material 1 [file 12284_2026_897_MOESM1_ESM.pdf]

Establishment of a non-transgenic iron-biofortified rice line using a novel *HRZ1* mutation

Authors:
Akihiro Saito[1]*, Junya Kumano[1], Masataka Suzuki[1], Kento Nakamura[1], Hiromi Ichinokawa[1], Arata Higashimoto[1], Mai Kato[1], Kei Shimada[1], Sachiho Koshika[1], Satomi Nakayama[1], Nanami Kawano[1], Shunta Nishino[1], Takehiro Kobayashi[1], Haruka Nakamura[1], Kurumi Yamanaka[1], Ayane Konno[1], Rina Shimokawa[1], Ryoma Sugano[1], Shuhei Mukaida[1], Hayate Hata[1], Takuji Ohyama[1], Yusuke Shikanai[1], Toshihiro Kumamaru[2], Shimpei Uraguchi[3], Toru Fujiwara[4], and Kyoko Higuchi[1]

Affiliations:
[1]Laboratory of Biochemistry in Plant Productivity, Department of Agricultural Chemistry, Tokyo University of Agriculture, Setagaya-ku, Tokyo 156-8502, Japan
[2]Faculty of Agriculture, Kyushu University, 744 Motooka Nishi-ku, Fukuoka, 819-0395, Japan
[3]Laboratory of Plant Nutrition, Graduate School of Horticulture, Chiba University, Matsudo-city, Chiba, 271-8510, Japan
[4]Laboratory of Plant Nutrition and Fertilizers, Department of Applied Biological Chemistry, Graduate School of Agricultural and Life Sciences, The University of Tokyo, Bunkyo-ku, Tokyo, 113-8657, Japan

Correspondence: a3saito@nodai.ac.jp

Supplementary data

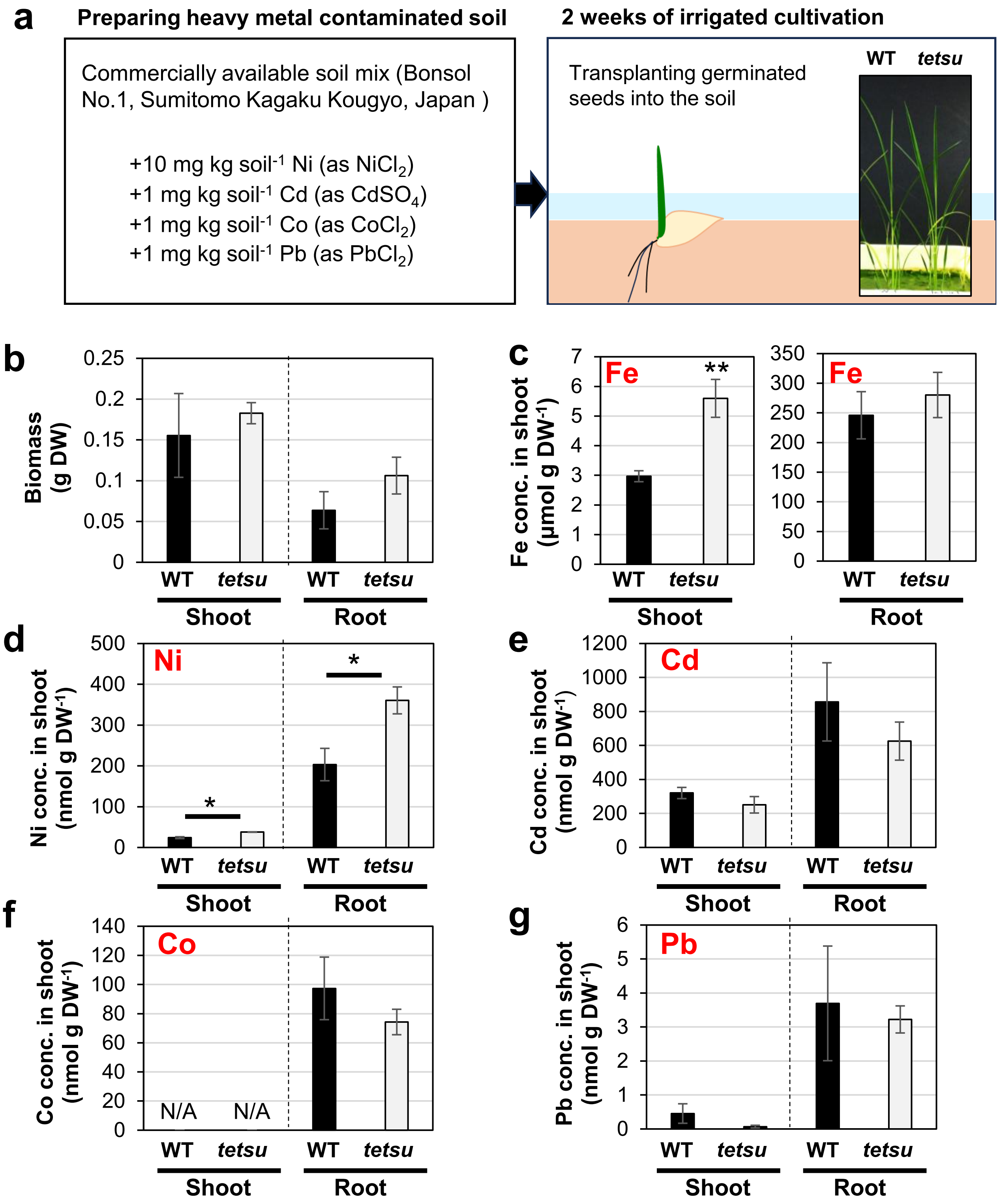

Figure S1. The *tetsu* mutant did not accumulate higher levels of toxic heavy metals in shoots compared with the wild type (WT)

(a) Schematic diagram of rice cultivation in heavy metal–contaminated soil.

(b) Dry weight of shoots and roots.

(c–g) Concentrations of Fe, Ni, Cd, Co, and Pb in shoots and roots of T65 and the *tetsu* mutant.

Seeds were sown immediately after germination into heavy metal–contaminated soil and grown for 14 d in a growth chamber at a light intensity of 250 μmol photons m^{-2} s^{-1} under a 14 h light (28 ° C)/10 h dark (24 ° C) photoperiod. Each column represents the mean ± SE (n = 4). Significant differences between T65 and tetsu were determined by Student's t-test (* $p < 0.05$, ** $p < 0.01$, n = 4).

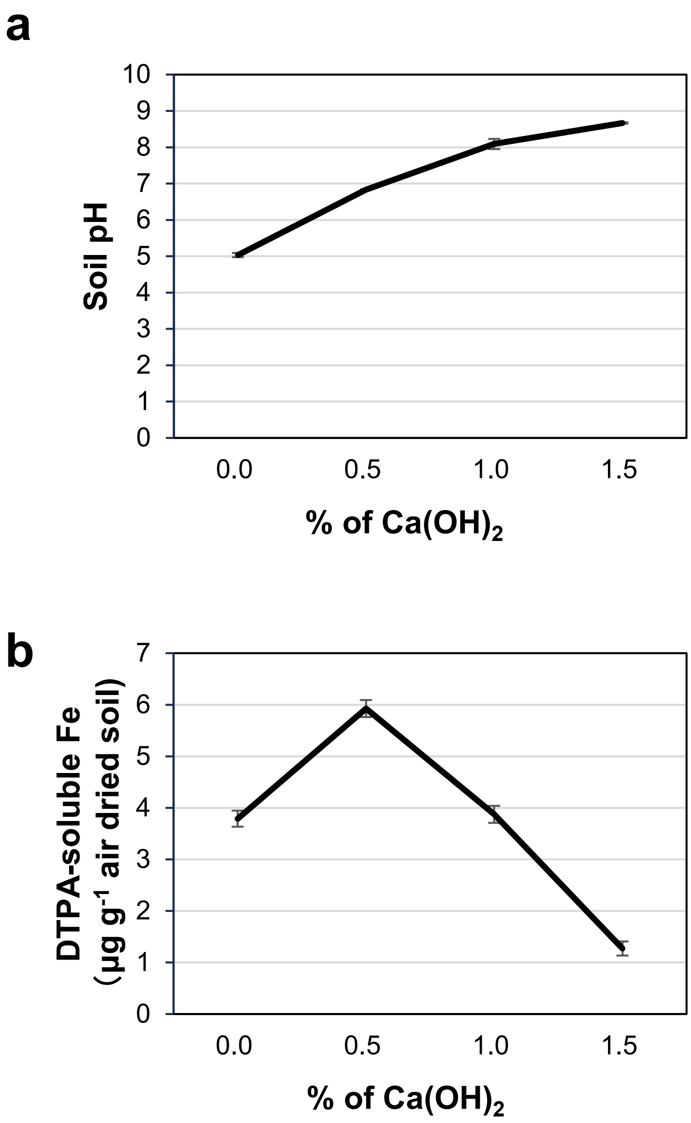

Figure S2. Alkalinization of cultivated soil by lime application and its effect on soluble Fe concentration
(a) Effect of calcium hydroxide concentration on the pH of the cultivated soil used in this study.
(b) Effect of calcium hydroxide concentration on the soluble Fe concentration in the cultivated soil used in this study (n = 3).

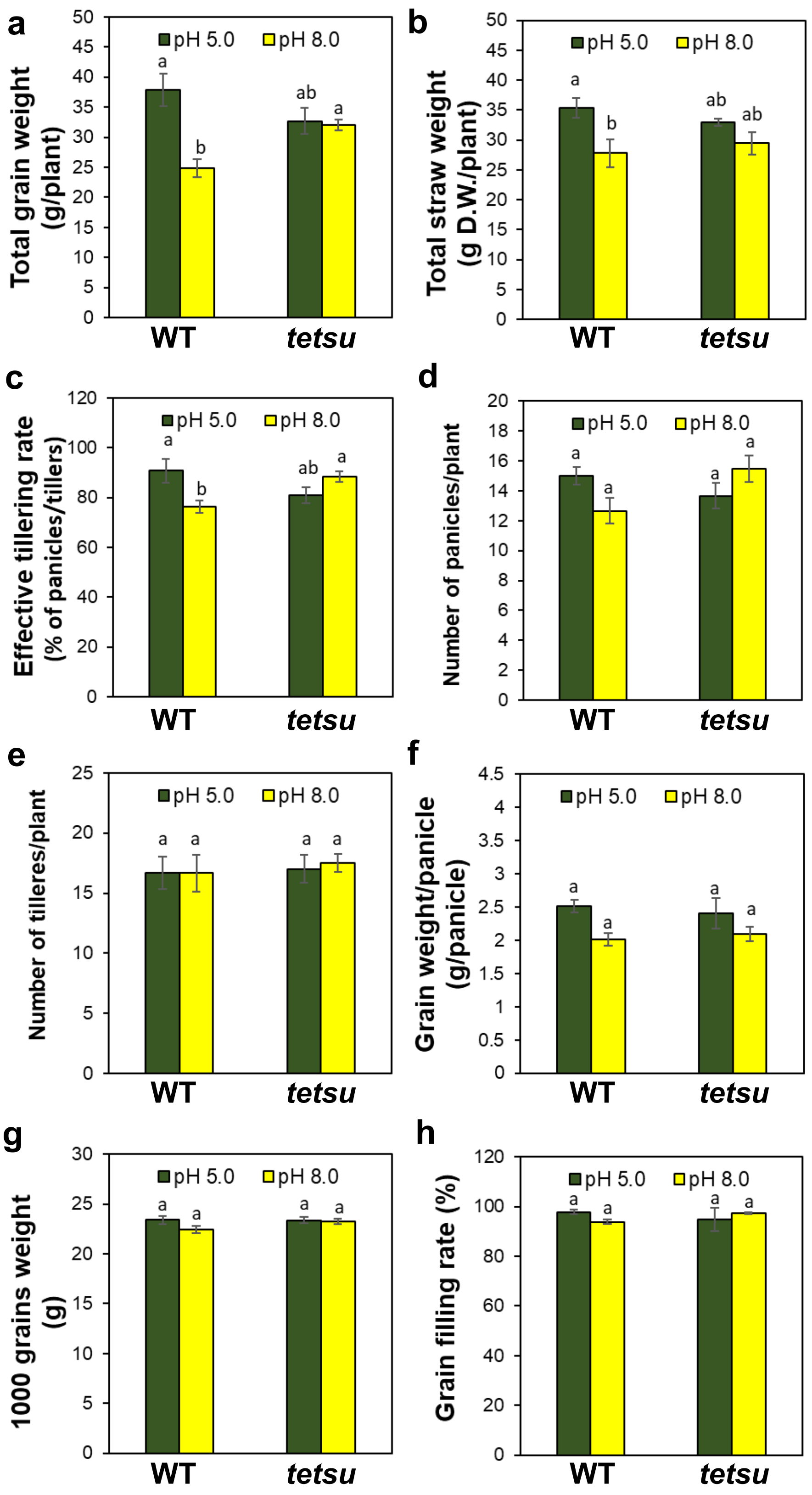

Figure S3. Yield survey of WT and the *tetsu* mutant grown in soil containing 1.0% $Ca(OH)_2$
The soil pH was adjusted to 8.0 by adding 1% calcium hydroxide to control soil with an initial pH of 5.0.
Other cultivation conditions were identical to those described in Fig. 3.
Data represent the mean ± SE (n = 3–6).
Means with the same letter are not significantly different at $p < 0.05$ according to Tukey–Kramer's multiple comparison test.

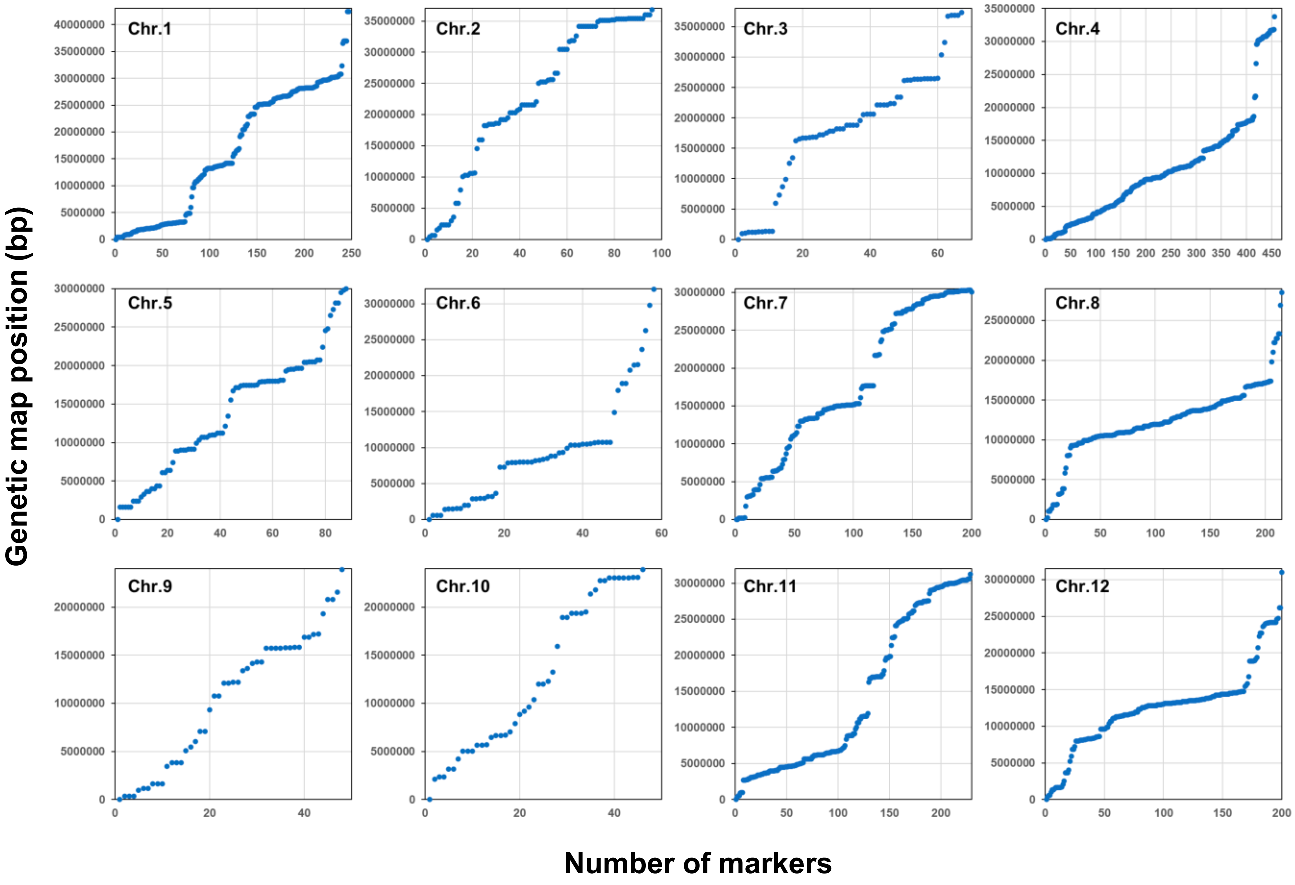

Figure S4. GRAS-Di markers cover the entire rice genome
Dot plots showing the relationship between marker number and genetic map position for each chromosome, indicating that the markers are widely distributed across the rice genome.

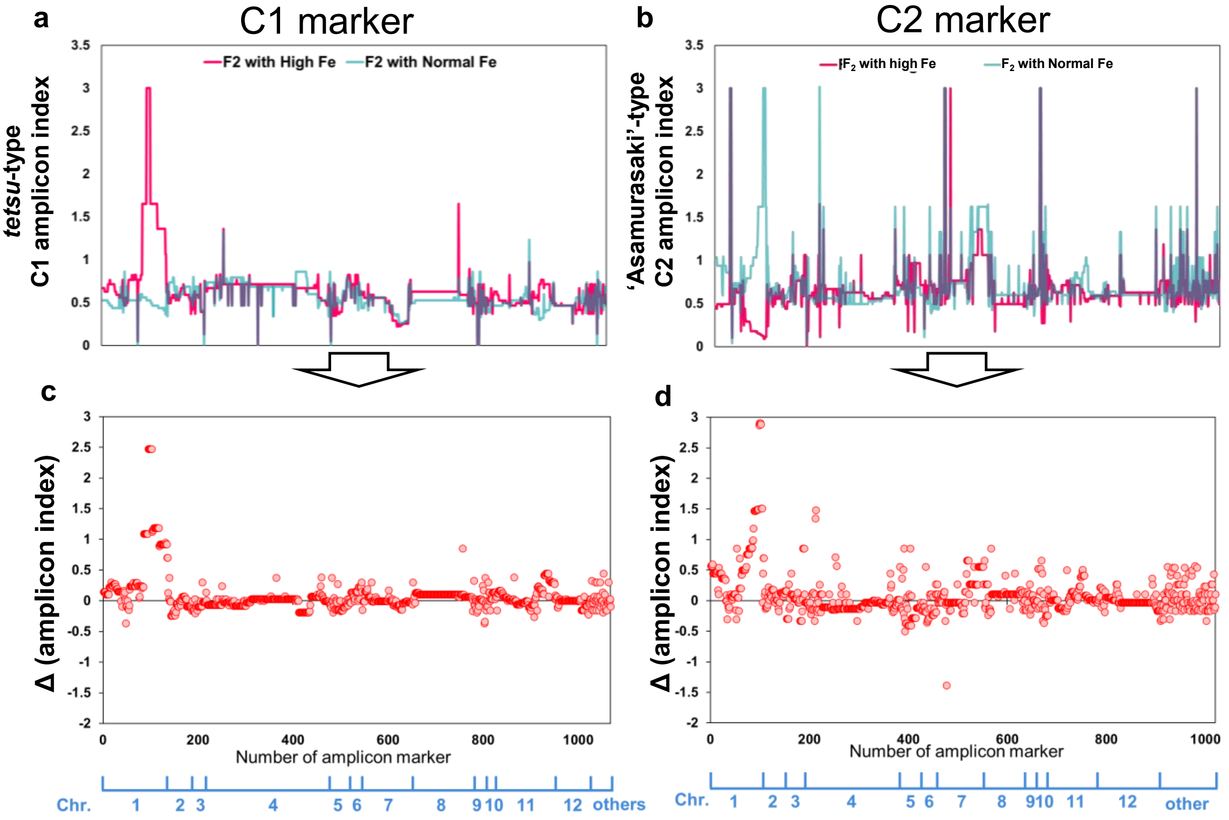

Figure S5. Two GRAS-Di markers identified a candidate region on chromosome 1 responsible for Fe hyperaccumulation in the *tetsu* mutant

To identify the causal loci for high Fe accumulation in the *tetsu*(*hrz1*) genome, the Δ (C1 amplicon index) for F_2 population with normal and high Fe levels was calculated according to the following formulas:

(1) Log{(1 – 'abundance ratio of the C1 marker in the F_2 population with normal Fe') + 0.001}
(2) Log{(1 – 'abundance ratio of the C1 marker in the F_2 population with high Fe') + 0.001}
(3) Δ (C1 amplicon index) = (2) – (1)

In formula (1), the "abundance ratio of the C1 marker in the F_2 population with normal Fe" represents the ratio of F_2 individuals with normal Fe that were positive for the C1 marker to the total of 50 F_2 plants with normal Fe. Similarly, in formula (2), the "abundance ratio of the C1 marker in the F_2 population with high Fe" represents the ratio of F_2 individuals with high Fe that were positive for the C1 marker to the total of 54 F_2 plants with high Fe. In formula (3), the Δ (C1 amplicon index) thus highlights the frequency of the C1 marker (dominant marker for *tetsu*) in the F_2 population with high Fe, with values ranging from a maximum of 3 to a minimum of 0.

The same calculation was performed for the C2 marker according to the following formulas:

(4) Log{(1 – 'abundance ratio of the C2 marker in the F_2 population with normal Fe') + 0.001}
(5) Log{(1 – 'abundance ratio of the C2 marker in the F_2 population with high Fe') + 0.001}
(6) Δ (C2 amplicon index) = (5) – (4)

(a) Individual amplicon indexes calculated using formulas (1) and (2).
(b) Individual amplicon indexes calculated using formulas (4) and (5).
The amplicon index for each F_2 population with high Fe content (red line) and normal Fe content (light blue line) is shown.
(c) Δ (amplicon index) based on the C1 marker.
(d) Δ (amplicon index) based on the C2 marker.
The Δ (C1 amplicon index) and Δ (C2 amplicon index) were combined into a single graph, as shown in Fig. 4d, where each marker was rearranged according to its chromosomal position.

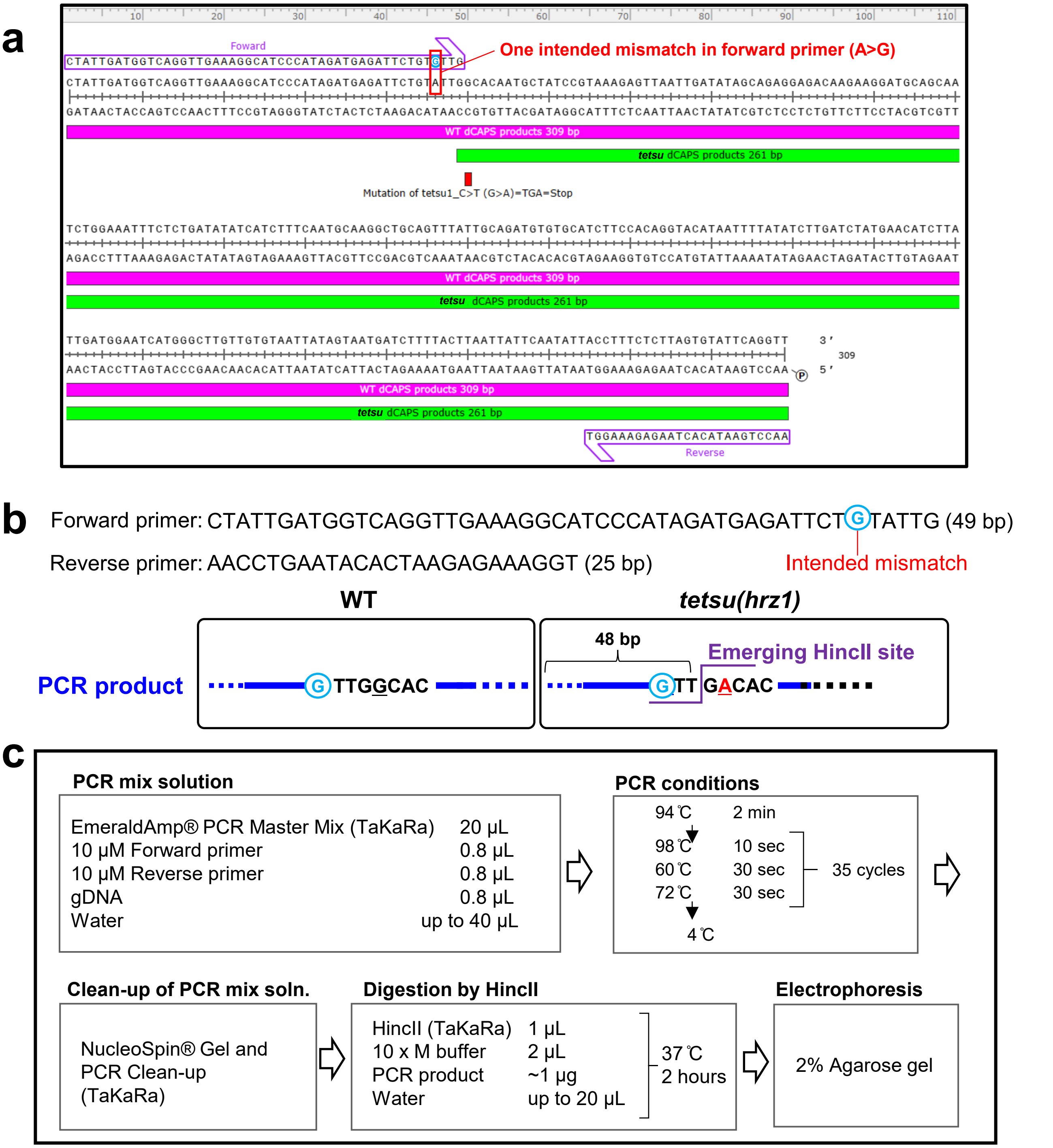

Figure S6. dCAPS analysis procedure for detecting the *tetsu* mutation in the *HRZ1* gene
(a) Sequence information of the forward and reverse primers (sequences in boxes indicated by arrows), and PCR products of the *HRZ1* gene from WT rice (magenta) and the *tetsu* mutant (green), displayed using SnapGene software (www.snapgene.com).
(b) Schematic representation of the site where the HincII restriction enzyme cleaves the PCR product.
(c) Details of the dCAPS experiment used to detect the *tetsu*(*hrz1*) mutation. PCR was performed using the EmeraldAmp® PCR Master Mix (RR300A, TaKaRa Bio Inc., Japan) on a thermal cycler (TP600TF, TaKaRa Bio Inc.) under the conditions described above. The resulting PCR products were purified using the NucleoSpin® Gel and PCR Clean-up kit (#740609.50, MACHEREY-NAGEL GmbH & Co.). Restriction digestion was performed in a reaction mixture containing HincII (1059A, TaKaRa Bio Inc., Shiga, Japan) at 37 ° C for 2 h according to the manufacturer's instructions. The digested products were electrophoresed on a 2% agarose gel, and the DNA bands were visualized by staining with DNA GelStain (RGS-50, RelyOn Ltd., Tokyo, Japan).

Table S1. List of mutations within the candidate locus on chromosome 1 in the *tetsu* mutant

Chr.No	Position	WT	*tetsu1*	Gene names with mutations
Chr.01	24249842	C	T	44 bp upstream of Os01g0612100 30S ribosomal protein S11, chloroplastic
Chr.01	24469050	C	T	No corresponding transcripts.
Chr.01	24487891	C	T	No corresponding transcripts.
Chr.01	24513239	C	T	No corresponding transcripts.
Chr.01	24513830	C	T	No corresponding transcripts.
Chr.01	24644694	C	T	No corresponding transcripts.
Chr.01	24715369	C	T	No corresponding transcripts.
Chr.01	24724477	C	T	No corresponding transcripts.
Chr.01	25316278	C	T	No corresponding transcripts.
Chr.01	25446043	C	T	No corresponding transcripts.
Chr.01	25578652	T	C	No corresponding transcripts.
Chr.01	25694011	C	T	No corresponding transcripts.
Chr.01	26022573	G	A	Transposon protein, putative, unclassified, expressed
Chr.01	26040405	G	A	No corresponding transcripts.
Chr.01	26061890	G	A	3'-UTR of Os01g0646700 (Similar to OSIGBa0105P02.4 protein)
Chr.01	26184578	G	A	No corresponding transcripts.
Chr.01	26527152	G	A	5'-UTR of Os01g0654200 Peptidase M50 domain containing protein.
Chr.01	26572245	G	A	Transposon protein, putative, unclassified, expressed
Chr.01	26894493	G	A	1st Intron of Os01g0660300 Similar to Pyruvate kinase.
Chr.01	27141209	G	A	Os01g0664300 Predicted gene
Chr.01	27262402	G	A	Missense mutation (Gly556>Asp556) in 7th Exon of Os01g0666800 Similar to predicted protein.
Chr.01	27273498	G	A	1st Intron of Os01g0667100 Similar to 60S ribosomal protein L18A.
Chr.01	27547056	C	T	No corresponding transcripts.
Chr.01	27642015	C	T	No corresponding transcripts.
Chr.01	27690308	C	T	No corresponding transcripts.
Chr.01	28428531	C	T	No corresponding transcripts.
Chr.01	28433699	C	T	No corresponding transcripts.
Chr.01	28455973	C	T	**Nonsense mutation (Trp301>Stop codon) in Os01g0689451 OsHRZ1** (Iron-binding haemerythrin RING ubiquitin ligase, Iron-binding sensor, Regulation of iron acquisition)
Chr.01	28808021	C	T	No corresponding transcripts.
Chr.01	28808494	C	T	No corresponding transcripts.
Chr.01	28915254	G	A	Missense mutation (Ser196>Ile196) in Os01g0698900 Similar to nascent polypeptide-associated complex alpha subunit-like protein.
Chr.01	29269967	T	C	No corresponding transcripts.
Chr.01	29468302	C	T	No corresponding transcripts.
Chr.01	29557909	C	T	No corresponding transcripts.
Chr.01	29684440	C	T	Os01g0714250 Predicted gene
Chr.01	29830275	C	T	5'-UTR of Os01t0716800-01 Endonuclease/exonuclease/phosphatase domain containing protein. phosphatidylinositol dephosphorylation
Chr.01	29957878	C	T	No corresponding transcripts.
Chr.01	29958349	C	T	No corresponding transcripts.
Chr.01	29991012	C	T	3rd intron of Os01t0719300-01 Sulfate transporter protein, Resistance to rice blast and bacterial leaf streak; Susceptibility gene

Table S2. Growth duration and developmental stages of parental lines and RILs under soil cultivation in Tokyo

Line	Genotype	Sowing date	Start of soil cultivation	Heading date	Grain filling period	Total cultivation days	Growth habit
Asamurasaki	Wild-type	17-May	22-May	29-Jul	Aug 3 – Sep 2	73	Early maturation
Taichung 65	Wild-type	17-May	22-May	21-Aug	Aug 29 – Oct 8	96	Late maturation
A-5BM	*tetsu* (*hrz1*)	17-May	22-May	14-Aug	Aug 18 – Sep 27	89	Mid maturation
C-1M	*tetsu* (*hrz1*)	17-May	22-May	7-Aug	Aug 14 – Sep 23	82	Mid-early maturation
A-3U	*tetsu* (*hrz1*)	17-May	22-May	10-Aug	Aug 14 – Sep 23	85	Mid-early maturation
